# Apposition of Fibroblasts With Metaplastic Gastric Cells Promotes Dysplastic Transition

**DOI:** 10.1053/j.gastro.2023.04.038

**Published:** 2023-08

**Authors:** Su-Hyung Lee, Ela W. Contreras Panta, David Gibbs, Yoonkyung Won, Jimin Min, Changqing Zhang, Joseph T. Roland, Se-Hoon Hong, Yoojin Sohn, Evan Krystofiak, Bogun Jang, Lorenzo Ferri, Veena Sangwan, Jiannis Ragoussis, Sophie Camilleri-Broët, Joseph Caruso, Chira Chen-Tanyolac, Michael Strasser, Philippe Gascard, Thea D. Tlsty, Sui Huang, Eunyoung Choi, James R. Goldenring

**Affiliations:** 1 Section of Surgical Sciences, Epithelial Biology Center, Vanderbilt University Medical Center, Nashville, Tennessee; 2 Department of Cell and Developmental Biology, Vanderbilt University, Nashville, Tennessee; 3 Institute for Systems Biology, Seattle, Washington; 4 Department of Pathology, Jeju National University School of Medicine, Jeju, Republic of Korea; 5 Division of Thoracic Surgery, Department of Surgery, McGill University, Montreal, Quebec, Canada; 6 McGill Genome Center, Department of Human Genetics, Victor Phillip Dahdaleh Institute of Genomic Medicine, McGill University, Montreal, Quebec, Canada; 7 Division of Thoracic Surgery, Department of Pathology, McGill University, Montreal, Quebec, Canada; 8 Department of Pathology, University of California San Francisco, San Francisco, California; 9 Nashville Veterans Affairs Medical Center, Nashville, Tennessee

**Keywords:** Gastric Carcinogenesis, Fibroblasts, Metaplasia, SPEM, PDGFRA

## Abstract

**Background & Aims:**

Elements of field cancerization, including atrophic gastritis, metaplasia, and dysplasia, promote gastric cancer development in association with chronic inflammation. However, it remains unclear how stroma changes during carcinogenesis and how the stroma contributes to progression of gastric preneoplasia. Here we investigated heterogeneity of fibroblasts, one of the most important elements in the stroma, and their roles in neoplastic transformation of metaplasia.

**Methods:**

We used single-cell transcriptomics to evaluate the cellular heterogeneity of mucosal cells from patients with gastric cancer. Tissue sections from the same cohort and tissue microarrays were used to identify the geographical distribution of distinct fibroblast subsets. We further evaluated the role of fibroblasts from pathologic mucosa in dysplastic progression of metaplastic cells using patient-derived metaplastic gastroids and fibroblasts.

**Results:**

We identified 4 subsets of fibroblasts within stromal cells defined by the differential expression of *PDGFRA*, *FBLN2, ACTA2*, or *PDGFRB.* Each subset was distributed distinctively throughout stomach tissues with different proportions at each pathologic stage. The PDGFRα^+^ subset expanded in metaplasia and cancer compared with normal, maintaining a close proximity with the epithelial compartment. Co-culture of metaplasia- or cancer-derived fibroblasts with gastroids showing the characteristics of spasmolytic polypeptide-expressing metaplasia–induced disordered growth, loss of metaplastic markers, and increases in markers of dysplasia. Culture of metaplastic gastroids with conditioned media from metaplasia- or cancer-derived fibroblasts also promoted dysplastic transition.

**Conclusions:**

These findings indicate that fibroblast associations with metaplastic epithelial cells can facilitate direct transition of metaplastic spasmolytic polypeptide-expressing metaplasia cell lineages into dysplastic lineages.

## Graphical abstract

**Figure:**
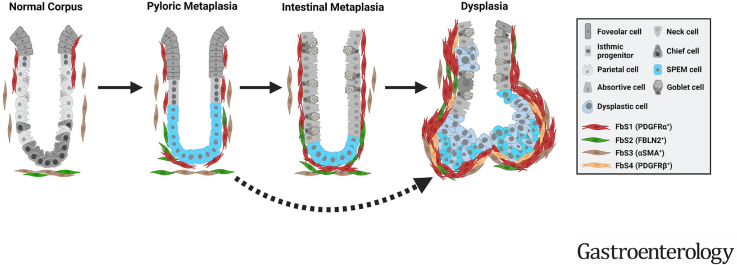

## Teaser

Altered stromal fibroblasts in the precancerous gastric lining promote the transition of precancerous metaplastic cells into dysplasia, supporting the development of gastric cancer.

**What You Need to Know**

**Background and Context**

Gastric cancer develops in a field of precancerous metaplasia and alterations in stromal elements. Little is known about the influence of fibroblast populations on the progression of metaplasia to dysplasia.

**New Findings**

Normal and pathologic gastric tissues showed 4 populations of fibroblasts, and co-culture of metaplasia-derived or cancer-derived fibroblasts with metaplastic gastroids induced progression to dysplasia.

**Limitations**

The mode of action for each fibroblast subset involved in precancerous progression needs to be clarified further.

**Clinical Research Relevance**

These investigations demonstrate that fibroblasts in the precancerous gastric mucosa can promote the development of dysplasia from metaplasia.

**Basic Research Relevance**

The results show that PDGFR ^+^ fibroblasts intimately associated with gastric metaplasia cells. Co-culture of metaplastic gastroids with the characteristics of spasmolytic polypeptide-expressing metaplasia with either metaplasia-derived or cancer-derived fibroblasts caused loss of metaplastic markers CD44v9 and AQP5 expression and gain of dysplasia markers TROP2 and CEACAM5 expression.

Intestinal-type gastric cancer usually develops through well-defined intermediate stages leading to carcinogenesis.^1^^,^^2^ Mucosal injury, characterized by loss of glandular elements, especially parietal cells, triggers chronic atrophic gastritis with alterations in immune cells and fibroblasts at the injured site, followed by sequential precancerous transitions, including metaplasia and dysplasia. The following 2 types of metaplasia have been identified in the corpus of the stomach: intestinal metaplasia (characterized by the presence of intestinal goblet cells)^3^ and pyloric metaplasia (characterized by the presence of spasmolytic polypeptide-expressing metaplasia [SPEM] lineage cells with features of deep antral gland cells).^4^ Intestinal metaplasia can be further subclassified as complete or incomplete, with incomplete intestinal metaplasia representing the highest risk for progression to cancer.^5^ Several studies have elucidated how normal stomach transitions to premalignancy in mouse models in response to genetic modification, pharmacologically induced parietal cell loss, or chronic *Helicobacter* spp infection.^6^, ^7^, ^8^

In most of the experimental models and patients with gastric cancer, interactions between elements of the microenvironment and epithelial cells seem to be crucial in disease initiation as well as progression. The microenvironment is composed of a field of heterogenous cell types, including immune cells, fibroblasts, and endothelial cells, which coordinate homeostasis in the normal gastric mucosa.^9^, ^10^, ^11^ Fibroblasts play an important role in establishing and organizing extracellular matrix, and are also a main source of soluble factors, such as WNT ligands, several cytokines and chemokines, and growth factors.^12^^,^^13^ Cancer-associated fibroblasts (CAFs) are thought to promote the expansion and migration of cancer cells in pancreatic ductal adenocarcinoma^12^ and breast cancer.^14^ Recent transcriptomic analyses at the single-cell level have revealed the cellular and molecular heterogeneity of epithelial and stromal lineages and the dramatic alterations in cell composition during gastric carcinogenesis in both humans and mice.^15^, ^16^, ^17^, ^18^ However, no previous studies have described the geographical distribution of diverse fibroblast subsets (FbSs) and their precise roles in development and progression of preneoplastic lesions in human stomach.

In this study, we analyzed mucosal cells from patients with gastric cancer. Based on single-cell RNA-sequencing (scRNA-seq) data analysis of fibroblasts from patients and immunostaining, 4 distinct FbSs were identified across normal, metaplasia, and dysplasia, or cancer with differential geographic distributions within tissues. We have also directly evaluated the role of fibroblasts in the progression of gastric preneoplasia using 2 different methodologies for 3-dimensional and Transwell air–liquid interface (ALI) co-cultures of metaplastic gastroids with fibroblasts isolated from tissues of patients with gastric cancer. These functional studies revealed that metaplasia- or cancer-derived fibroblasts promote metaplasia progression to dysplasia.

## Methods

### Ethics Declaration

The research protocol governing management and stewardship of patient tissue detailed in this article was approved by the McGill University Health Care Research Ethics Board (2007-856). All patient information was anonymized and deidentified before experiments and the clinicopathologic information, such as tumor grade and clinical stage, is provided in Supplementary Table 1.

### Bioinformatic Methods

The entire workflow was implemented using Singularity containers for reproducibility (all codes available, please contact authors for information).

### Three-Dimensional Co-Culture and Transwell Air–Liquid Interface Co-Culture

For 3-dimensional co-culture in Matrigel, separately maintained fibroblasts and gastroids were collected as described above and suspended in 30 μL of Matrigel/type I collagen mixture. Approximately 60,000 fibroblasts in 15 μL of Matrigel/collagen mixture were mixed with small chunks of gastroids in 15 μL of Matrigel/collagen mixture. A total 30 μL of final mixture per well was seeded on a 48-well plate for live imaging or an 8-well chamber slide for whole-mount staining, and then 300 μL of IntestiCult medium was added to each well after polymerization of Matrigel/collagen mixture for 30 minutes at 37°C. Phase-contrast live imaging was performed on the JuLI stage, a Real-Time Cell History Recorder (NanoEntek), and an EVOS M7000 inverted microscope (ThermoFisher).

ALI cultures were performed in 24-well plate Transwells (Corning). Transwell filters were previously coated by adding 300 μL of type I collagen (2.7 mg/cm^2^) and incubating for 30 minutes at 37°C. After the incubation, the remaining collagen liquid was removed to air dry the filters. Fibroblasts derived from normal, metaplasia, or cancer were harvested and seeded (approximately 60,000 cells in 200 μL of fibroblast medium) on the collagen-coated filters. Three hundred microliters of fibroblast medium were added to the well under the Transwell filter. Fibroblasts were incubated at 37°C, 5% CO_2_ in a humidified incubator for 6 hours. During that time, gastroids were incubated with Organoid Harvesting Solution (R&D Systems) for 30 minutes at 4°C to remove Matrigel (ECM; Corning). After incubation, gastroids were centrifuged and the supernatant was removed. The pellet was resuspended in TrypLE (Gibco) to obtain single-cell suspensions. A total of 90,000 cells of gastroids in approximately 200 μL of human IntestiCult medium (StemCell Technology) were seeded onto the top of a collagen-coated Transwell filter with or without fibroblasts (gastroid only). Fibroblast medium under the Transwell filter was replaced with 300 μL of human IntestiCult medium. Top and bottom IntestiCult medium were changed after 48 hours. Seeded gastroid cells were left for 7 days to grow on top of collagen-coated Transwells layered with or without fibroblasts. On day 7, the media overlying the cells were removed to expose the surface of the cells to air and the ALI process lasted 14 days. The media at the bottom wells were replaced every 3 days and fluid secreted from the cells was removed every day from the top Transwells.

Further detailed methods are included in the Supplementary Methods.

## Results

### Single-Cell RNA Sequencing Reveals Cellular Heterogeneity of Human Gastric Mucosal Cell Lineages

To profile cellular and molecular heterogeneity during gastric carcinogenesis, we performed scRNA-seq on the adjacent normal (gastritis), metaplastic, dysplastic, or cancerous stomach tissues from 5 patients with gastric cancer. We analyzed a total of 72,126 cells after quality control from freshly prepared tissues, based on the following lineage-specific genes: *CLDN18* and *EPCAM* for gastric epithelial cells; *PTPRC* (encoding CD45; total hematopoietic cells), *CD3D* (T cells), *CD79A* (B cells), *CSF1R* (macrophages), *CSF3R* (granulocytes), and *TPSAB1* (mast cells) for immune cells; *COL1A1* and *ACTA2* for fibroblasts and myofibroblasts; *vWF* and *PECAM1* for vascular endothelial cells; and *NRXN1* and *GPM6B* for neural cells (Supplementary Figure 1*A–G*).

We next examined subsets within gastric epithelial cells. As shown in Supplementary Figure 2*A* and *B* and Supplementary Figure 3*A, CLDN18*- and/or *EPCAM*-expressing epithelial clusters in the Uniform Manifold Approximation and Projection of total cells were further divided into 9 different groups with several undefined clusters, including gastric, intestinal, and dysplastic or cancer cell lineages. We also identified a dual *AQP5*^*+*^*/MUC6*^+^ SPEM-lineage population in cluster 2, a major component of pyloric metaplasia, as reported previously.^7^ Notably, compared with the chief cell cluster (cluster 3), transdifferentiating (cluster 16) and SPEM cells in cluster 2 displayed graded decreases in expression of *PGA3*, *PGA4*, and *PGA5* with sequential increases of *AQP5*, *MUC6*, and *CLU* (Supplementary Figure 2*C*). Intestinal metaplasia lineage clusters showed the characteristics of normal intestinal cell types, including *VIL1*^+^ absorptive cells (cluster 6), *DMBT1*^+^ intestinal metaplastic intermediate cells (clusters 5 and 7), *TFF3*^*+*^ and/or *MUC2*^+^ goblet cells (cluster 18), and 2 different types of enteroendocrine cells (clusters 12 and 13), but *DEFA5*^+^ Paneth cells were rarely observed (Supplementary Figure 3*A*). In addition, we identified a distinctive intestinal cell lineage purely composed of *CFTR*^+^ and *BEST4*^+^ cells (cluster 21), known to reside in the villus of human small intestine, but whose function is not well-understood (Supplementary Figure 3*A*).^19^^,^^20^ Compared with other lineages, the intestinal metaplasia clusters 5, 6, 7, and 18 highly expressed *DMBT1*, *CLDN7*, *MUC17*, *ANPEP*, *REG4*, *MUC3A*, *KRT20*, *PHGR1*, *CDHR2*, and *CDHR5* (Supplementary Figure 2*C*).

### Dysplastic Cell Lineages Specifically Express CEACAM5

To understand the molecular heterogeneity of the cells at the final step in gastric carcinogenesis and identify potential markers for dysplastic cell lineages, we focused on clusters 11 and 14, which were designated as dysplastic or cancer cell clusters on the basis of the expression of *TACSTD2*, the transcript for TROP2 protein (Supplementary Figure 2*C* and Supplementary Figure 3*B*).^21^ These 2 clusters were also enriched with *OLFM4*, *CLDN3*, *CLDN4*, *EPCAM*, *CD9*, *MUC13*, *AQP3*, *CEACAM5*, and *CEACAM6* (Supplementary Figure 2*C* and Supplementary Figure 3*B*). A previous report described increased expression of CLDN4 during gastric carcinogenesis.^22^ We observed that *CLDN3*, *CLDN4*, and *CLDN7* transcripts were up-regulated in most of the dysplastic or cancer cells, although these genes were also expressed in some of the intestinal cell lineages (Supplementary Figure 2*C* and Supplementary Figure 3*B* and *C*). In addition, we found several markers that can more specifically define dysplastic or cancer clusters. Matrix metallopeptidase 7 has been implicated in invasion and metastasis of tumor cells and is mainly expressed in epithelial cells.^23^ Accordingly, *MMP7* transcripts in the present data set very specifically marked a portion of cluster 11 (Supplementary Figure 3*C*). *CEACAM5* and *CEACAM6* identically defined the remaining portion of cluster 11 and the whole cluster 14 (Supplementary Figure 3*C*).

We next evaluated these markers for dysplastic lineages at the protein level in tissue microarrays containing 24 cores, including the following 36 regions of interest in human early gastric cancer resections: 5 regions for adjacent normal, 7 regions for incomplete intestinal metaplasia, 11 regions for dysplasia, and 13 regions for intestinal-type gastric cancer. Although TROP2, CLDN7, and CEACAM5 were completely negative in adjacent normal regions, more than one-half of the regions of incomplete intestinal metaplasia, dysplasia, and gastric cancer expressed TROP2 (71.4%, 54.5%, and 53.8%, respectively) and/or CLDN7 (85.7%, 63.6%, and 63.6%, respectively) (Supplementary Figure 2*D* and *E*). CEACAM5, known as a marker indicating poor prognosis of gastric cancer,^24^ was weakly expressed in only 28.6% of incomplete intestinal metaplasia regions. However, in accordance with our transcriptomic data, dysplastic and cancerous cells more strongly and frequently showed apical CEACAM5 expression (90.9% and 76.9%, respectively) (Supplementary Figure 2*D* and *E*).

### Single-Cell RNA Sequencing Identifies Distinct Fibroblast Subsets at Each Stage of Gastric Carcinogenesis

**Figure 1:**
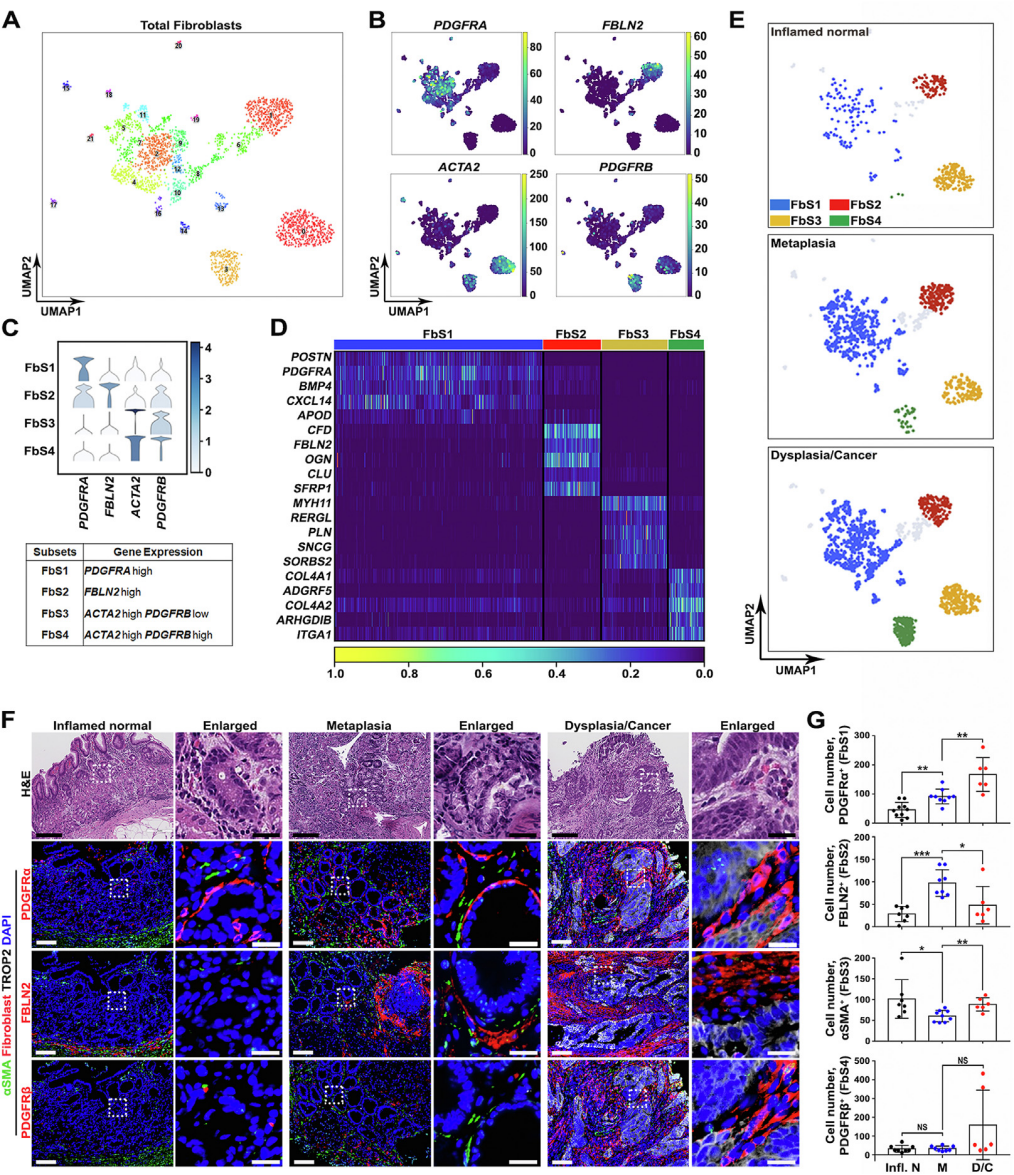
scRNA-seq defines 4 different fibroblasts subsets (FbSs) with a distinct geographic distribution. (*A*) Uniform Manifold Approximation and Projection (UMAP) of 2709 fibroblasts in 21 color-coded clusters. Each *dot* in the UMAP indicates an individual cell. (*B*) UMAPs representing expression of selected markers for fibroblasts and myofibroblasts. Based on the marker genes, the total fibroblast population can be divided into 4 subsets. (*C*) *Violin plo*t of normalized expression of *PDGFRA*, *FBLN2*, *ACTA2*, and *PDGFRB* in the different subsets; *PDGFRA*^hi^ FbS1, *FBLN2*^hi^ FbS2, *ACTA2*^hi^*PDGFRB*^lo^ FbS3, and *ACTA2*^hi^*PDGFRB*^hi^ FbS4. (*D*) *Heatmap* of selected genes enriched in each of the 4 FbSs according to scRNA-seq data. *Columns* indicate single cells. (*E*) *UMAPs* color-coded according to pathologic condition, based on the expression of *PDGFRA*, *FBLN2*, *ACTA2*, and *PDGFRB*. Representative *images* (*F*) and quantification (*G*) of immunofluorescence staining for PDGFRα for FbS1, FBLN2 for FbS2, αSMA for FbS3, PDGFRβ for FbS4, and dysplastic marker TROP2 with nuclear 4′,6-diamidino-2-phenylindole (DAPI) in gastric cancer patient–derived tissue sections of inflamed normal, metaplasia, or dysplasia/cancer regions. Data are presented as mean ± SD (n = 6–10 tissue sections of 7 patients). ∗*P* < .05; ∗∗*P* < .01; ∗∗∗*P* < .001. *Scale bar:* 100 μm and 20 μm for enlarged.

We analyzed the transcriptomic profiles of fibroblasts to identify whether distinct subsets exist in the stomach, as described in pancreas or breast tissues.^25^^,^^26^ Unsupervised clustering resolved a total of 21 fibroblast clusters that could be assigned to 4 large subsets—FbS1, *PDGFRA*^hi^; FbS2, *FBLN2*^hi^; FbS3, *ACTA2*^hi^ *PDGFRB*^lo^; and FbS4, *ACTA2*^hi^ *PDGFRB*^hi^ (Figure 1*A–C*)—which were consistently observed in different pathologic conditions (Supplementary Figure 4*A* and *B*). Although FbS1 and FbS2 were linked by Uniform Manifold Approximation and Projection analysis of the scRNA-seq results, the FbS3 and FbS4 cells defined clearly separate clusters (Figure 1*A* and *B*). Notably, each subset had a distinctive gene signature (Figure 1*D* and Supplementary Figure 4*C*). *POSTN* and *TGFBI*, encoding important extracellular matrix components during inflammation, were abundant in FbS1 (Supplementary Figure 4*C* and *D*). *IL1R1,* which encodes the interleukin (IL) receptor type 1 responsible for proinflammatory IL1β signaling, was mostly enriched in FbS1, but less abundant in FbS2 and FbS4 (Supplementary Figure 4*D*). In our dataset, gene expression of various cytokines and chemokines typically up-regulated in inflammation was not significantly detected. However, some cells of FbS1 and FbS2 were enriched in *CXCL14*, *CCL2*, *CCL11*, *CXCL12*, or *IL33* transcripts (Supplementary Figure 4*D*). Other inflammatory response-related genes, such as *SERPINF1*, *FCGRT*, and *DCN*, were also mainly expressed in FbS1 and FbS2 (Supplementary Figure 4*D*).

Gene Ontology analysis of differentially expressed genes also revealed that each FbS is involved in different biological processes during gastric carcinogenesis (Supplementary Figure 5*A*). The differentially expressed genes in the *PDGFRA*^hi^ subset (FbS1) were enriched in pathways related to extracellular matrix organization and cell motility (Supplementary Figure 5*A*). The differentially expressed genes in the *FBLN2*^hi^ subset (FbS2) were uniquely enriched in complement- or neutrophil-associated inflammatory pathways (Supplementary Figure 5*A*). The *ACTA2*^hi^ *PDGFRB*^lo^ subset (FbS3) demonstrated the characteristics of myofibroblasts, while the *ACTA2*^hi^ *PDGFRB*^hi^ FbS4 subset had a gene signature consistent with a pericyte population, including increased expression of *ACTA2*, *RGS5*, *NDUFA4L2*, *CSPG4, NOTCH3*, and *ENPEP* (Supplementary Figures 4*C* and *D* and 5*A*).

Because fibroblast populations are key sources of WNT ligands,^27^ we also sought to evaluate the expression of WNT signaling-related genes in the 4 subsets. FbS1 was the main source of *BMP4*, *WNT4*, and *WNT5A*, whereas FbS2 highly expressed *WNT2B* and *RSPO3* compared with other subsets (Supplementary Figure 5*B* and *C*). WNT pathway-associated gene expression was rare in FbS3, and FbS4 expressed *WNT6* and *BMP8A* (Supplementary Figure 5*B* and *C*).

Pathologic diagnosis-based analysis revealed that the proportion of FbS2 and FbS3 was most abundant in the non-metaplastic inflamed mucosa with normal lineages (subsequently referred to as inflamed normal) and decreased in metaplasia and dysplasia or cancer (Figure 1*E*). PDGFRα, encoded by *PDGFRA*, is a well-known marker for CAFs in pancreas and has been used for identification of telocytes in the intestine.^26^^,^^28^ Interestingly, the proportion of *PDGFRA*^hi^ FbS1 cells increased in more advanced lesions compared with the inflamed normal (Figure 1*E*), implying that expansion of FbS1 may be associated with progression of gastric carcinogenesis. In contrast, the FbS4 pericyte population was only prominently observed in dysplastic or cancer samples (Figure 1*E*).

### Each Fibroblast Subset Is Differentially Distributed Throughout Stomach Tissues

**Figure 2:**
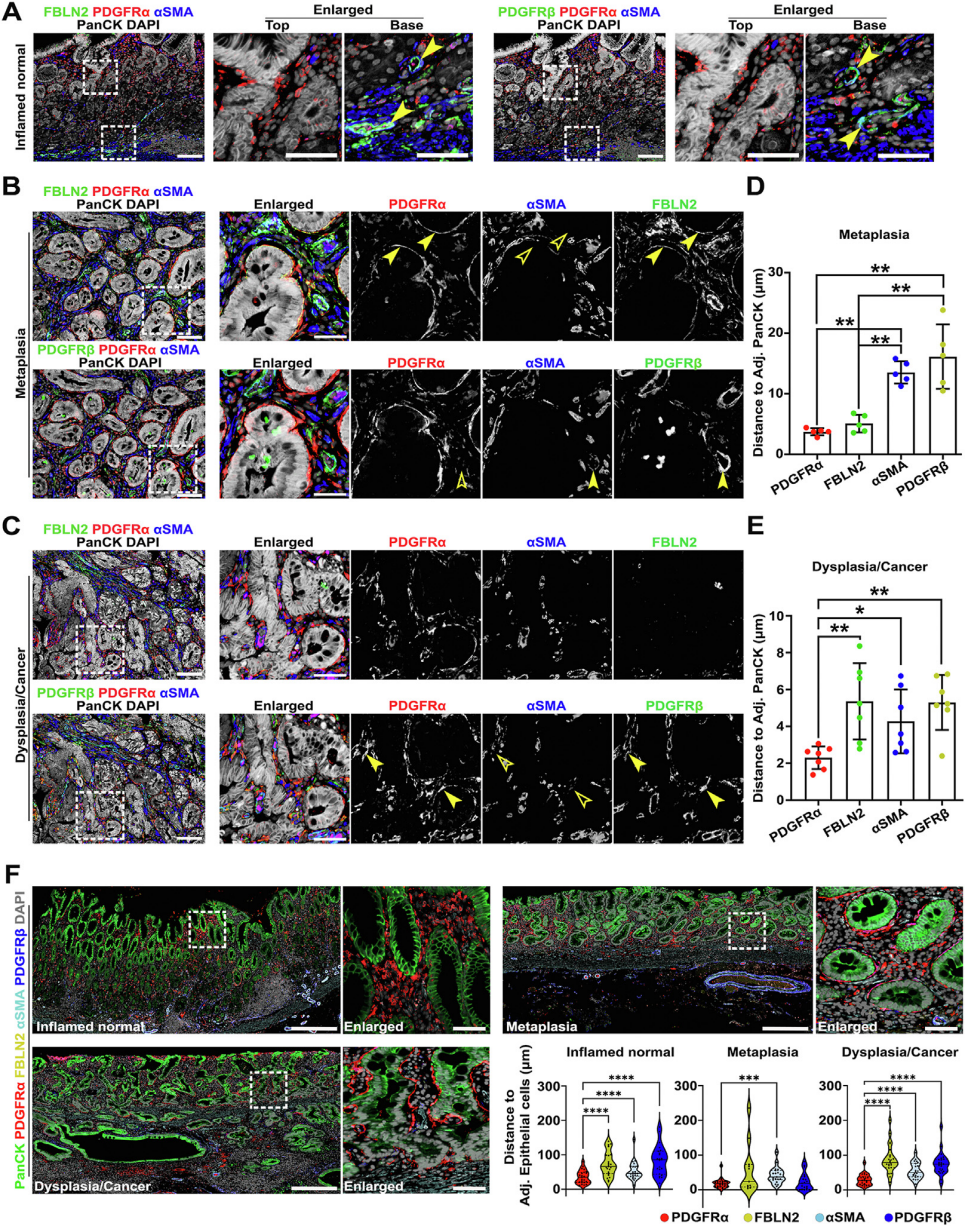
Multiplexed immunofluorescence staining revealed geographic distribution of FbSs during gastric carcinogenesis. (*A–C*) Representative *images* of immunofluorescence staining for PDGFRα for FbS1, FBLN2 for FbS2, αSMA for FbS3, PDGFRβ for FbS4 fibroblasts, and epithelial marker pan-cytokeratin (PanCK) with nuclear 4′,6-diamidino-2-phenylindole (DAPI) in human gastric cancer patient*–*derived tissue sections. (*A*) Inflamed normal tissue. PDGFRα^+^ cells are present in the isthmus region of the corpus mucosa. *Arrowheads* indicate αSMA^+^ vascular structure with FBLN2 (*left panel*) or PDGFRβ (*right panel*) expression. (*B*) Metaplastic tissue. PDGFRα^+^ cells are observed in close apposition to metaplastic gland cells. *Arrowheads* in the *upper panel* indicate fibroblasts with strong PDGFRα^+^ staining and weaker FBLN2^+^ without αSMA expression (*empty arrowheads*) surrounding metaplastic glands. *Arrowheads* in the *lower panel* indicate αSMA^+^PDGFRβ^+^ vascular structures without PDGFRα expression (*empty arrowheads*). (*C*) Cancer tissue. PDGFRα^+^ cells are expanded between cancerous glands. *Arrowheads* in the *lower panel* indicate PDGFRα^+^PDGFRβ^+^ fibroblasts surrounding cancerous epithelial compartment without αSMA expression (*empty arrowheads*). *Scale bar:* 100 μm and 50 μm for enlarged. (*D, E*) Quantification of the distance between epithelial cells and neighboring FbSs in metaplastic (*D*) and cancer-bearing stomach tissues (*E*). Data are presented as mean ± SD (n = 5 patients). ∗*P* < .05; ∗∗*P* < .01. (*F*) Representative *images* and quantification of multiplexed immunofluorescence staining for PDGFRα, FBLN2, αSMA, and PDGFRβ cells and their relationship with epithelial cells (positive for PanCK, CD44v9, or TROP2) with nuclear 4′,6-diamidino-2-phenylindole (DAPI) staining from 3 human tissue microarray slides (n = 24 cores per each pathologic condition). ∗∗∗*P* < .001; ∗∗∗∗*P* < .0001. *Scale bar:* 500 μm and 50 μm for enlarged.

Based on the transcriptomic data, we investigated geographical distribution and population changes of FbSs during carcinogenesis through immunostaining for subset-specific markers. In the adjacent normal gastric corpus tissues with or without inflammation, PDGFRα^+^ FbS1 mostly resided in the isthmus region surrounding corpus glands (Figures 1*F* and 2*A*). Notably, this subset was morphologically similar to telocytes in the intestine, with elongated extension of cytoplasmic processes.^27^ In metaplastic tissues, FbS1 fibroblasts were prominently expanded throughout entire metaplastic glands in close proximity to the metaplastic lineages, often observed cupping SPEM cells at the bases of metaplastic glands (Figures 1*F* and *G* and 2*B*). FBLN2 expression was occasionally observed in the PDGFRα-expressing cells (Figure 2*B*). The PDGFRα^+^ FbS1 in dysplastic or cancerous tissues also wrapped around the epithelial compartment, but with a more disorganized pattern (Figures 1*F* and *G* and 2*C*). In contrast to FbS1 in metaplastic tissues, in dysplasia or cancer regions, these cells frequently co-expressed PDGFRβ (Figure 2*C*). FBLN2^+^ FbS2 fibroblasts in the adjacent normal and metaplastic tissues were mainly observed in the submucosa associated with inflammatory cell infiltration or vascular structures (Figures 1*F* and 2*A*). In more severely inflamed metaplastic lesions, this subset expanded around adjacent metaplastic lineages, as shown for FbS1, but was slightly set back from the gland compared with FbS1 (Figures 1*F* and 2*B*). Although the FbS2 population was considerably enhanced in the metaplastic tissues compared with inflamed normal tissues (Figure 1*G*), this subset exhibited variation in the cell number in the dysplastic or cancer tissues, depending on the extent of inflammation (Figures 1*G* and 2*C*).

α–Smooth muscle actin ([αSMA]; encoded by *ACTA2*)-positive FbS3 myofibroblasts were mostly present in the submucosa of the inflamed normal, metaplastic, and dysplastic/cancer tissues (Figure 1*F* and Figure 2*A–C*). Although the distribution pattern of this subset was not significantly different among most of the samples, cell numbers were slightly decreased in the metaplastic stomachs (Figure 1*G*). Furthermore, there were no significant differences in PDGFRβ^+^ FbS4 pericytes throughout most of the samples, although a few cancerous tissues showed a prominent expansion of the FbS4 population with or without PDGFRα co-expression surrounding the epithelial compartment (Figure 1*F* and *G* and Figure 2*A–C*).

We also examined the geographic association of FbSs with epithelial cells. Although FbS1 and FbS2 surrounded metaplastic glands, FbS3 and FbS4 were located significantly farther from the metaplastic epithelial cells (Figure 2*D*). In the dysplastic or cancer tissues, most of the fibroblasts were closer to the epithelial compartment compared with the subsets in the metaplastic tissues (Figure 2*E*). In particular, PDGFRα^+^ fibroblasts with or without expression of other markers were positioned adjacent to epithelial cells compared with other FbSs (Figure 2*E*). To confirm these relationships, we also examined 3 human tissue microarrays with samples of gastritis, metaplasia, or dysplasia or early gastric cancer using multiplexed immunostaining and unbiased digital quantitation and analysis. Figure 2*F* demonstrates that, as seen for the original patients from McGill, PDGFRα^+^ fibroblasts showed significantly closer apposition to epithelial cells compared with αSMA^+^ myofibroblasts in all samples.

### Fibroblasts Derived From Different Lesions in a Human Gastric Cancer Patient Stomach Have Distinctive Characteristics

**Figure 3:**
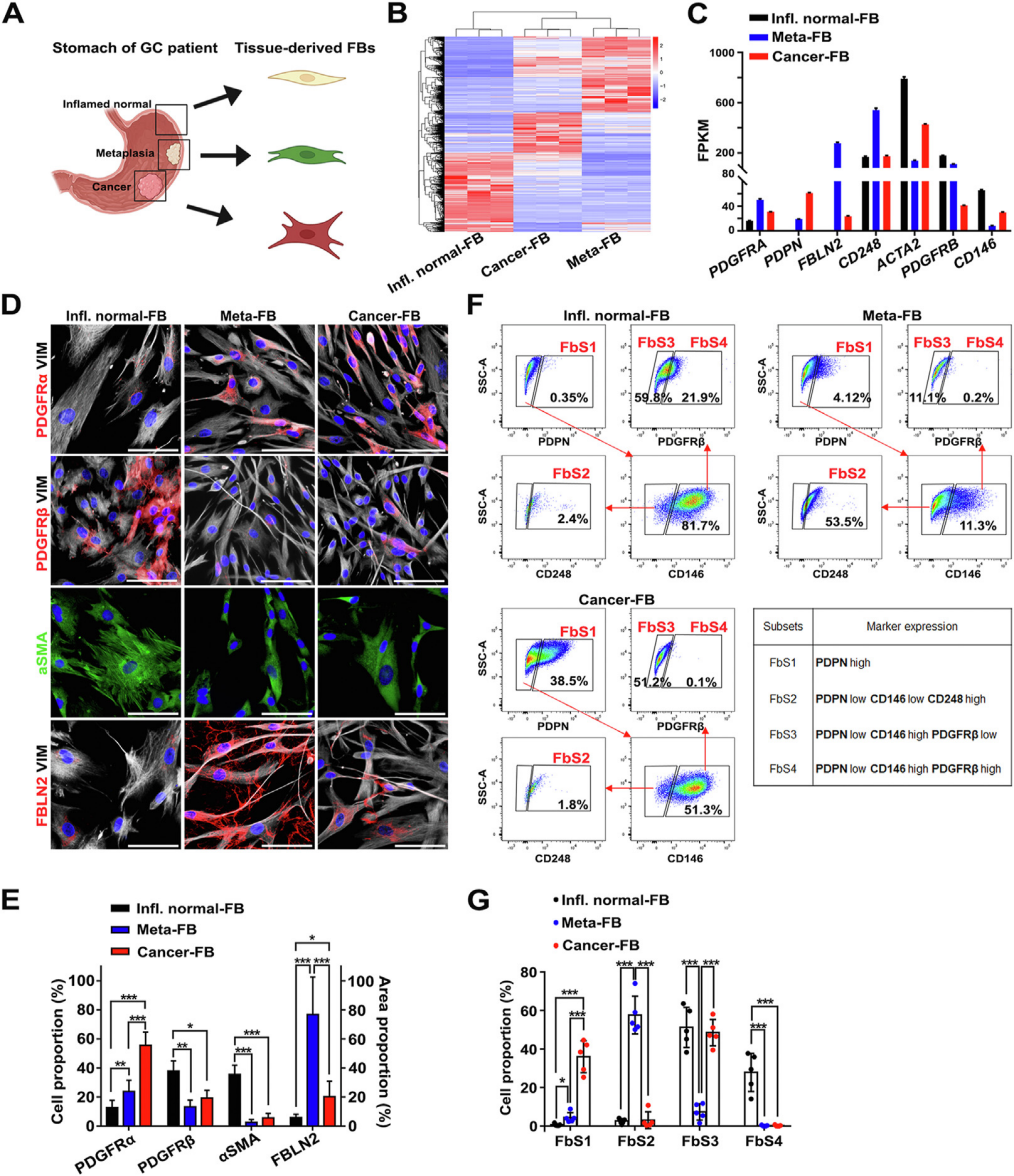
Specific pathologic condition-derived fibroblasts (FBs) have distinctive genetic characteristics and cell composition. (*A*) *Schematic illustration* of FB isolation from human gastric cancer (GC) patient samples. (*B*) *Heatmap* of differentially expressed genes (DEGs) in inflamed normal- (Infl. Normal-FB), metaplasia- (Meta-FB), and cancer-derived FBs (Cancer-FB). *Rows* and *columns* represent individual genes and replicates per each group, respectively. (*C*) *Bar graph* representing the normalized counts of selected marker genes in different lesion-derived FBs, confirmed by scRNA-seq data from human patient samples. Representative *images* (*D*) and quantification (*E*) of immunofluorescence staining for the markers for each FB subset in different lesion-derived FBs. Data are presented as mean ± SD (n = 4 independent experiments). ∗*P* < .05; ∗∗*P* < .01; ∗∗∗*P* < .001. *Scale bar:* 50 μm. Fluorescence-activated cell sorting *plots* showing the proportion of 4 FB subsets, described in the table, in different lesion-derived FB populations (*F*) and quantification (*G*). Data are presented as mean ± SD (n = 5 independent experiments). ∗*P* < .05; ∗∗∗*P* < .001.

To investigate the role of fibroblasts in progression of gastric preneoplastic lesions, we freshly isolated and established gastroid and fibroblast lines from inflamed normal, metaplasia, and cancerous tissues of human patients (Figure 3*A*) and profiled their phenotypic and genetic features. First, bulk RNA-seq of 3 fibroblast lines derived from inflamed normal, metaplastic, or cancer-bearing tissues demonstrated that each line had a quite different gene signature (Figure 3*B* and Supplementary Figure 6*A–C*). Although the inflamed normal-derived fibroblasts prominently expressed *ACTA2*, *PDGFRB*, and *CD146,* which were hallmarks of FbS3 myofibroblasts and FbS4 pericytes in the scRNA-seq data analysis, the metaplasia-derived fibroblasts were enriched instead for genes up-regulated in FbS2, such as *FBLN2* and *CD248*. Of note, the cancer-derived fibroblasts had significantly increased expression of *PDGFRA* and *PDPN* compared with inflamed normal-derived cells, indicating these fibroblasts were transcriptionally similar to FbS1 (Figure 3*C*). Compared with inflamed normal-derived fibroblasts, genes up-regulated in the metaplasia- or cancer-derived fibroblasts were enriched for “channel activity,” “receptor-ligand activity,” and “extracellular matrix structural constituent” pathways (Supplementary Figure 6*D* and *E*). These pathway-related gene signatures were further up-regulated in cancer-derived fibroblasts compared with metaplasia-derived fibroblasts (Supplementary Figure 6*F*).

We also examined protein expression levels for markers of each FbS in patient-derived fibroblasts. In inflamed normal-derived fibroblasts, almost 40% of the cells showed positivity for PDGFRβ or αSMA, whereas PDGFRα- or FBLN2-expressing cells were rare (Figure 3*D* and *E*). The metaplasia-derived fibroblasts were enriched in intracellular and extracellular FBLN2, and 20% of the cells were positive for PDGFRα (Figure 3*D* and *E*). As shown in the transcriptome analysis, cancer-derived fibroblasts showed an increased proportion of PDGFRα-expressing cells compared with other fibroblasts. While seen more prominently in metaplasia-derived fibroblasts, some of the fibroblasts from cancer-bearing mucosa also showed intracellular and extracellular FBLN2 positivity (Figure 3*D*).

To identify the composition of patient-derived fibroblast populations more precisely, we next examined expression of several surface markers (podoplanin [PDPN], CD248, CD146 [MCAM] and PDGFRβ) through fluorescence-activated cell sorting, using a gating strategy based on the transcriptomic data analyses (Supplementary Figure 7*A* and *B*). PDPN is one of the major surface markers for CAFs in pancreatic cancer.^12^ We confirmed that *PDPN* was uniquely enriched in the FbS1 cluster. The proportion of PDPN^hi^ FbS1 fibroblasts was increased 10- or 100-fold in metaplasia- or cancer-derived fibroblasts, respectively, compared with inflamed normal-derived fibroblasts (Figure 3*F* and *G*). Although the inflamed normal-derived fibroblasts were enriched for PDPN^lo^ CD146^hi^ FbS3 and FbS4 cells, more than one-half of the metaplasia-derived fibroblasts were PDPN^lo^ CD146^lo^ CD248^hi^ FbS2 cells (Figure 3*F* and *G*), as observed with the level of *CD248* transcript expression and FBLN2 protein staining.

### Isolation of Patient-Derived Gastroids With the Characteristics of Spasmolytic Polypeptide-Expressing Metaplasia

For characterization of gastroids derived from metaplastic tissues of 2 different patients, immunostaining for several lineage-specific markers was performed. The tissue sections-derived from cancerous or adjacent noncancerous regions of patients 1–3 demonstrated morphologic features of inflamed normal, metaplastic, or dysplastic or cancer in histopathologic examination (Supplementary Figure 8*A*). The gastroids demonstrated phenotypic features similar to the tissue of origin with positivity for SPEM (CD44v9, AQP5) and/or gastric antral and intestinal lineage markers (PDX1 and CDX1). However, expression of TROP2 or CEACAM5, markers for more advanced preneoplastic lesions, such as incomplete intestinal metaplasia or dysplasia (Supplementary Figure 8*B*), was weak or rare in these gastroids (Supplementary Figure 8*C*). These staining data indicated that the gastroids established from patient tissues were SPEM-lineage predominant metaplastic gastroids.^7^

### Co-Culture of Metaplastic Gastroids With Human Metaplasia- or Cancer-Derived Fibroblasts Promotes Progression Into Dysplasia

**Figure 4:**
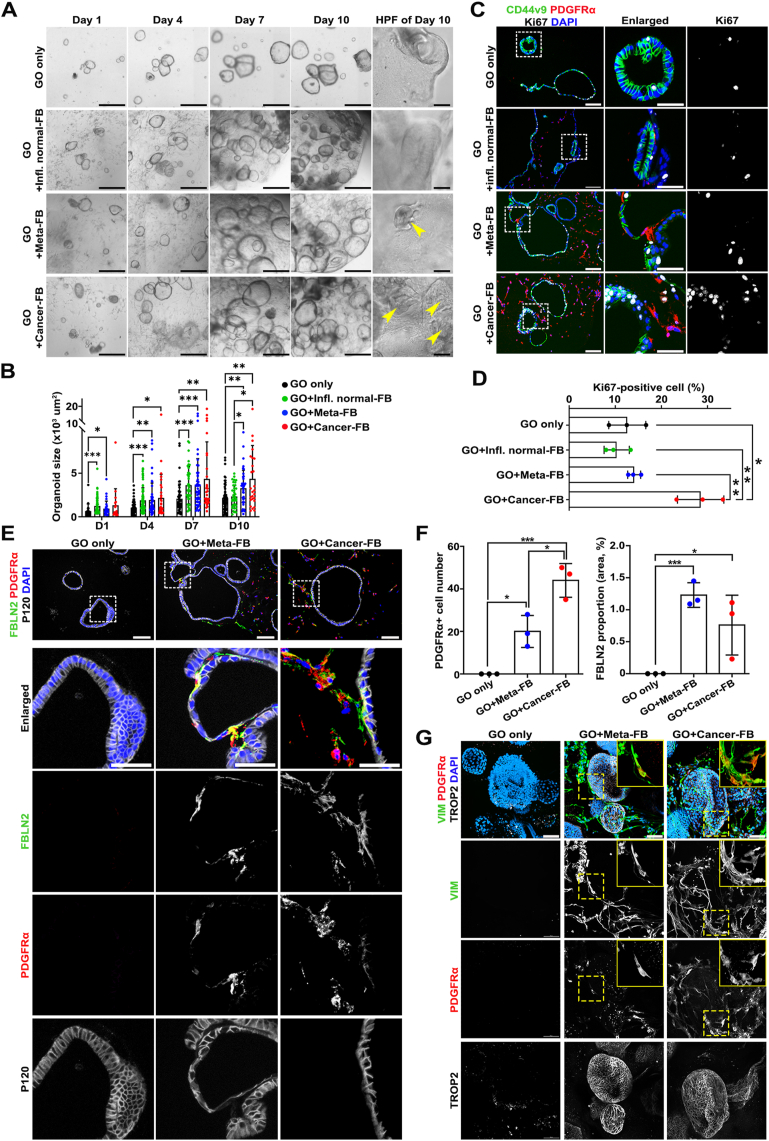
Three-dimensional co-culture with metaplasia-derived and cancer-derived fibroblast (FB) enhances growth of metaplastic gastroids. (*A*) Representative *brightfield images* of gastroids (GOs) cultured for 10 days with or without FBs isolated from inflamed normal-, metaplastic-, or cancer-bearing mucosae. *Arrowheads* indicate budding formation. *Scale bar:* 500 μm and 100 μm for high-power field. (*B*) Quantification of GO size at each time point. Data are presented as mean ± SD (n = 20–90 organoids from 2 independent experiments). ∗*P* < .05; ∗∗*P* < .01; ∗∗∗*P* < .001. (*C*) Representative *images* of immunofluorescence staining for metaplasia marker CD44v9, PDGFRα for FbS1, and Ki67 for proliferative activity with nuclear 4′,6-diamidino-2-phenylindole (DAPI). (*D*) Quantification of proliferative GO cells. Data are presented as mean ± SD (n = 3 independent experiments). ∗*P* < .05; ∗∗*P* < .01. Representative *images* (*E*) and quantification (*F*) of immunofluorescence staining for FBLN2 for FbS2, PDGFRα for FbS1 and epithelial membrane marker P120 with nuclear DAPI. Data are presented as mean ± SD (n = 3 independent experiments). ∗*P* < .05; ∗∗∗*P* < .001. (*G*) Representative *images* of whole-mount staining for vimentin (VIM) for pan-FBs, PDGFRα for FbS1, and dysplasia marker TROP2 with nuclear DAPI. *Boxes* are enlarged *insets* showing VIM^+^PDGFRα^+^ FbS1 close by GOs.

Based on these characteristics of fibroblasts and gastroids, we first performed a 3-dimensional co-culture of the metaplastic gastroids with metaplasia- or cancer-derived fibroblasts to investigate whether gastroid phenotypes can be altered by different types of fibroblasts. Initially, fibroblasts were irregularly distributed in Matrigel domes with gastroids, but began to migrate toward the gastroids at day 3, and some finally were situated close to gastroids, similar to the pattern seen in human tissues (Figure 4*A*, *C*, and *E*). After 7 days, gastroids co-cultured with either metaplasia- or cancer-derived fibroblasts grew faster than gastroids cultured alone and reached maximal size (Figure 4*A* and *B*). However, proliferative activity was significantly enhanced by co-culture with cancer-derived fibroblasts, but not with inflamed normal- or metaplasia-derived fibroblasts (Figure 4*C* and *D*). We next sought to examine the cell composition of fibroblasts and gastroids after co-culture. In accordance with profiles of patient-derived fibroblasts cultured alone, metaplasia- or cancer-derived fibroblasts in co-culture with gastroids remained mostly positive for PDGFRα and FBLN2 and abundantly expressed extracellular transforming growth factor–β-induced (TGFBI), whereas inflamed normal-derived fibroblasts in co-culture mainly consisted of αSMA^+^ cells (Figure 4*E* and *F* and Supplementary Figure 9*A* and *B*). Furthermore, cancer-derived fibroblasts showed a 2-fold higher number of PDGFRα^+^ fibroblasts compared with metaplasia-derived fibroblasts, and intra- or extracellular FBLN2 positivity was slightly up-regulated in metaplasia-derived fibroblasts compared with cancer-derived fibroblasts (Figure 4*F*).

In terms of geographical correlation between fibroblasts and gastroids, while αSMA^+^ cells from inflamed normal-derived fibroblasts were irregularly scattered between gastroids, several PDGFRα^+^, FBLN2^+^, and/or TGFBI^+^ cells from metaplasia- or cancer-derived fibroblasts directly contacted with gastroids (Figure 4*E* and Supplementary Figure 9*A* and *B*), similar to FbS1 and FbS2 that closely surrounded epithelial compartments in various preneoplastic lesions of human stomach (Figure 1*F* and Figure 2*A–C*). Some of the PDGFRα^+^ cancer-derived fibroblasts surrounding gastroids also expressed PDGFRβ (Supplementary Figure 9*B*), as observed in fibroblasts adjacent to epithelial cells in cancer tissues. Whole-mount staining also revealed that a portion of VIM^+^ fibroblasts with extended cytoplasmic processes came directly in contact with gastroids (Figure 4*G* and Supplementary Figure 9*C*). Given that FbS1 fibroblasts were enriched for genes involved in “cell migration,” these data suggest that the different FbSs might display distinct migratory activity toward epithelial cells. As shown in paraffin section staining, PDGFRα^+^ fibroblast populations were more prominently observed in cancer-derived fibroblasts compared with metaplasia-derived fibroblasts in 3-dimensional whole-mount immunostaining (Supplementary Figure 9*C*).

**Figure 5:**
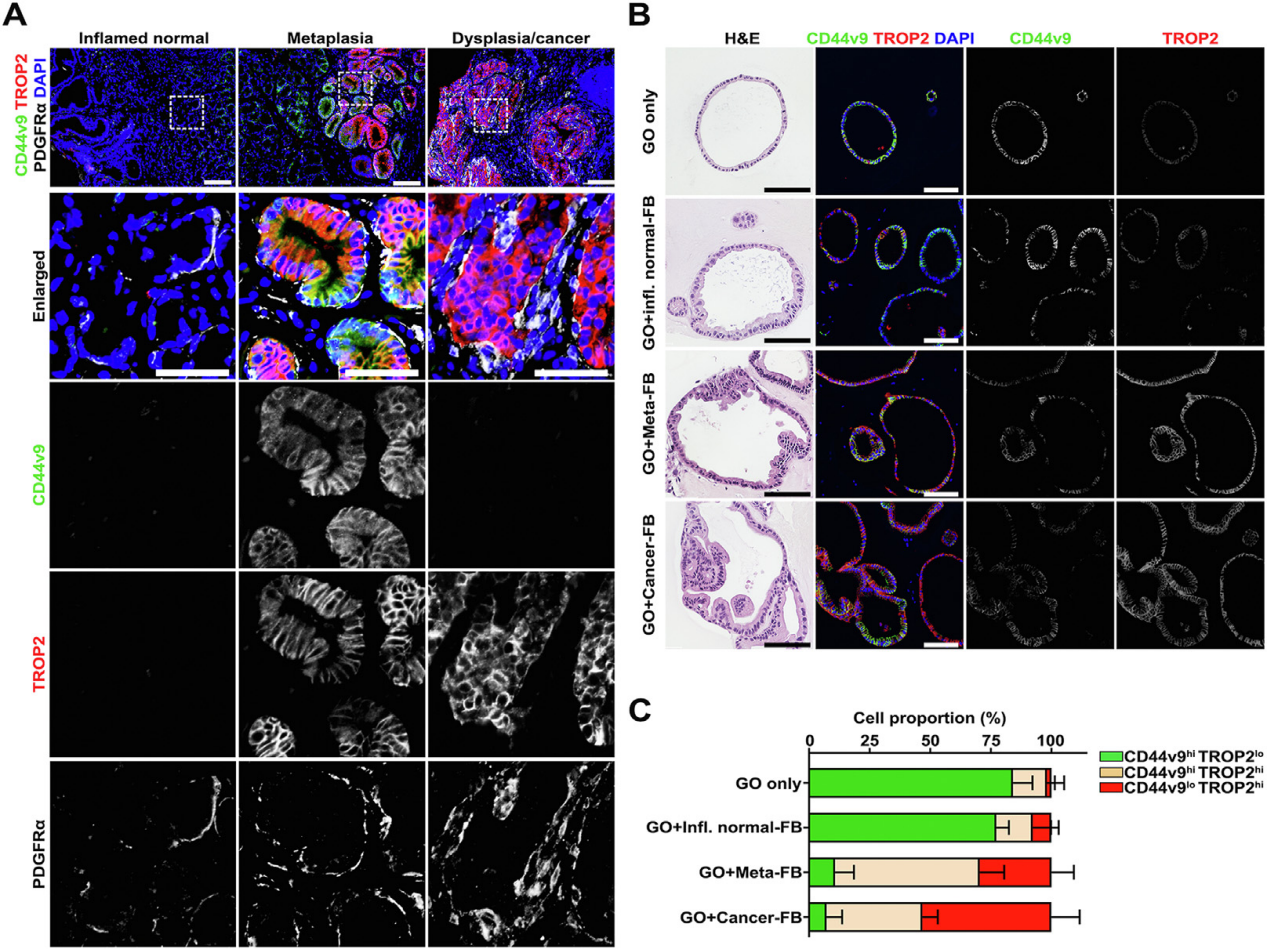
Recruited metaplasia-derived and cancer-derived fibroblasts facilitate dysplastic progression in metaplastic gastroids (GOs). (*A*) Immunofluorescence staining in human stomach sections for metaplasia marker CD44v9, dysplasia marker TROP2, and FbS1 marker PDGFRα with nuclear 4′,6-diamidino-2-phenylindole (DAPI). *Scale bar:* 100 μm and 50 μm for enlarged. Representative *images* of immunofluorescence staining in GO sections for metaplasia marker CD44v9 and dysplasia marker TROP2 (*B*) and quantification of positive GO cells for each marker (*C*). *Scale bar:* 100 μm.

We previously reported that TROP2 is a marker for incomplete intestinal metaplasia and dysplasia, and that CD44v9 is a metaplasia marker in stomach mucosa.^21^^,^^29^ In the present study, we found differential expression of CD44v9 and TROP2 in metaplastic or dysplastic or cancer tissues. As shown in Figure 5*A*, adjacent normal tissues with or without inflammation did not express either CD44v9 or TROP2, whereas metaplastic glands were positive for CD44v9 and/or TROP2. Of note, dysplastic/cancer tissues showed only TROP2 expression. Taken together, altered expression of CD44v9 and TROP2 in a gland can therefore be considered as a hallmark of dysplastic transition in metaplastic glands.^21^ Accordingly, almost 80% of cells in cultured metaplastic tissue*–*derived gastroids strongly expressed CD44v9 with low TROP2 levels. Although inflamed normal-derived fibroblasts did not induce alterations in marker expression, the proportion of TROP2-expressing cells with similar or lower CD44v9 expression increased by almost 90% in gastroids co-cultured with metaplasia- or cancer-derived fibroblasts (Figure 5*B* and *C*).

### Direct Contact With Metaplasia- or Cancer-Derived Fibroblasts Induces Dysplastic Transition in Metaplasia

**Figure 6:**
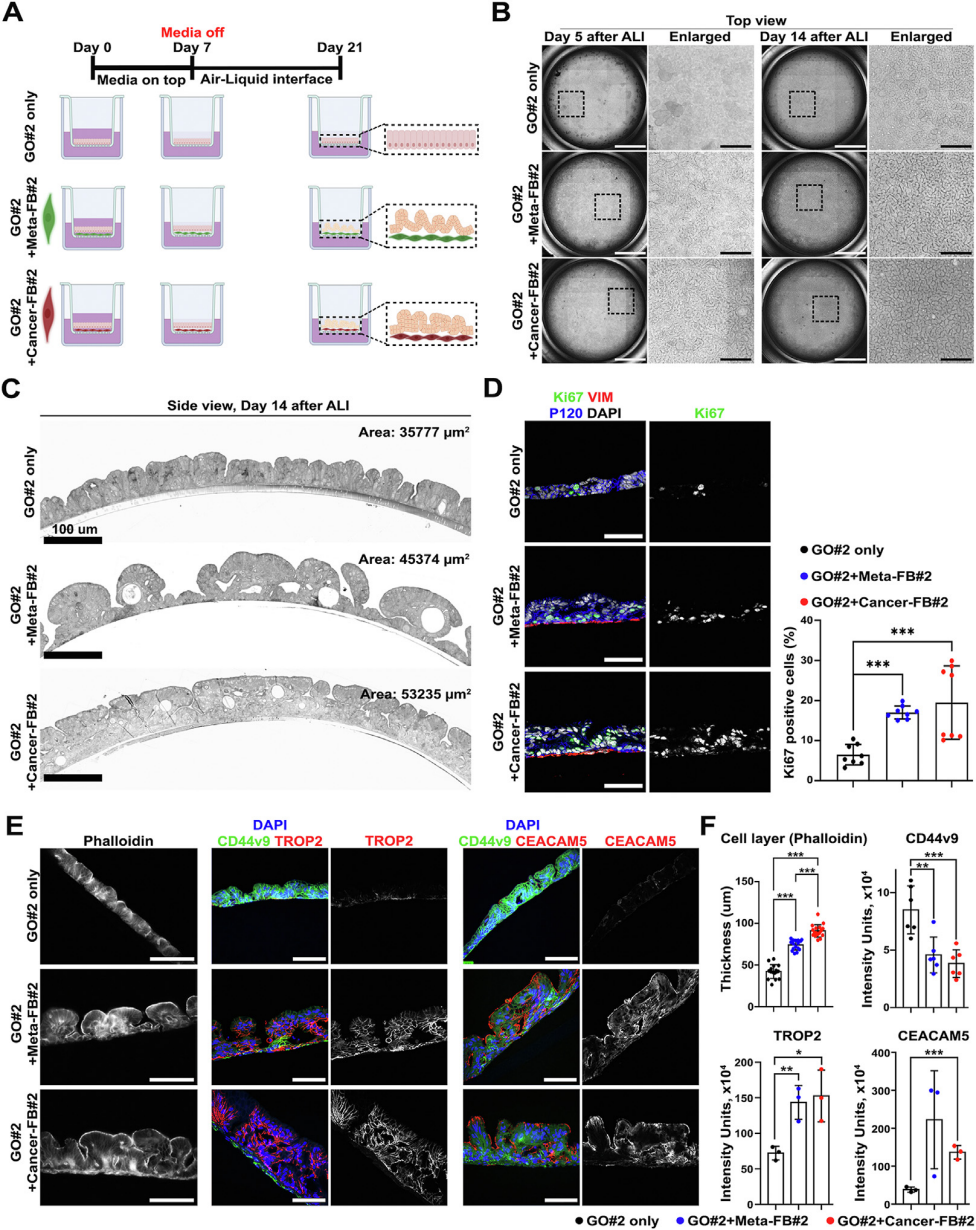
ALI co-culture demonstrates that metaplasia (Meta)- or cancer-derived fibroblasts (FBs) can induce dysplastic transition in precancerous metaplastic cells. (*A*) *Schematic illustration* of ALI co-culture of metaplastic gastroids (GOs) with Meta- or cancer-derived FBs for a total of 21 days (created with BioRender.com). Representative *brightfield images* (*top view; B*) and *confocal images* of plastic sections stained with toluidine blue (*side view; C*) of ALI co-culture of metaplastic GO cells with Meta- or cancer-derived FBs, both from patient 2. *Scale bar:* 2000 μm and 500 μm for enlarged. Note the multiple-layered disorganized cells after co-culture with Meta- or cancer-derived FBs in (*C*). (*D*) Representative *images* of immunofluorescence staining for proliferation marker Ki67, fibroblast marker vimentin (VIM), and epithelial membrane marker P120 with nuclear 4′,6-diamidino-2-phenylindole (DAPI) and quantification of Ki67-positive cells. Data are presented as mean ± SD (n = 8 images from 2 different sections). ∗∗∗*P* <.001. (*E*) Representative *images* of immunofluorescence staining for phalloidin to identify cell borders, metaplasia marker CD44v9, and dysplasia markers TROP2 or CEACAM5 with nuclear DAPI. *Scale bar:* 100 μm. Note the loss of CD44v9 and the gain of TROP2 and CEACAM5 expression when GO cells were cultured in the presence of Meta- or cancer-derived FBs. (*F*) Quantification of the thickness of GO cell layer determined by phalloidin staining and extent of protein marker expression measured by intensity units. Data are presented as mean ± SD (n = 5 or 3 of different sections for phalloidin or other markers, respectively). ∗*P* < .05; ∗∗*P* < .01; ∗∗∗*P* < .001.

To facilitate the direct interaction of fibroblasts in contact with metaplastic gastroid cells, we employed an ALI co-culture system on Transwell filters composed of metaplastic gastroids from patient 2 with metaplasia- or cancer-derived fibroblasts from the same patient (Figure 6*A*). At day 5 after media removal for initiation of the ALI condition, gastroid cells without fibroblasts predominantly displayed a single layer in the center of the well (Figure 6*B*). Interestingly, gastroid cells co-cultured with either metaplasia- or cancer-derived fibroblasts for 5 days after ALI initiation formed large numbers of protrusions from the monolayer throughout the well and maintained polypoid structures at day 14 (Figure 6*B*). At this time point, projecting polyps of co-cultured gastroid cells covered the entire area of the well and coalesced with each other, resulting in a “gyri and sulci-like structure” (Figure 6*B* and *C*). Accordingly, proliferative activity in gastroid cells increased in co-culture conditions with either metaplasia- or cancer-derived fibroblasts compared with gastroids cultured without fibroblasts (Figure 6*D*).

We next investigated CD44v9, TROP2, and CEACAM5 expression in gastroid cells in each condition to evaluate whether the morphologic changes induced by co-culture with fibroblasts correlated with metaplasia progression. Metaplastic gastroid cells cultured alone mostly displayed strong CD44v9 expression (Figure 6*E* and *F*). In contrast, co-culture with metaplasia- or cancer-derived fibroblasts enhanced TROP2 expression in cells within polypoid projections, whereas CD44v9 expression was rarely observed or showed residual expression at the basal cell layer (Figure 6*E* and *F*). We then evaluated CEACAM5 expression in these gastroid cells based on the findings that CEACAM5 was prominently and specifically expressed in dysplastic tissues compared with metaplastic tissues (Supplementary Figure 2*D* and *E* and Supplementary Figure 8*A*). Gastroid cells strongly expressed CEACAM5 only after co-culture with either metaplasia- or cancer-derived fibroblasts, especially in the polypoid regions (Figure 6*E* and *F* and Supplementary Figure 10*A–C*). Finally, metaplastic gastroid cells derived from patient 1 or 2 were co-cultured with metaplasia- or cancer-derived fibroblasts from different patients (patient 1, 2, or 3) to assess whether these effects of fibroblasts on epithelial cells were restricted to co-culture with the same patient-derived cells. All patient-derived fibroblasts from either metaplasia- or cancer-bearing tissues also induced polypoid protrusions of metaplastic gastroid cells from the Transwell membranes (Supplementary Figure 11*A* and *C*).

Expression of the metaplasia marker CD44v9 was more intensely and consistently observed in the gastroid cells without fibroblasts than in co-cultured gastroid cells (Supplementary Figure 11*B* and *D*). The SPEM cell lineage-specific marker AQP5 was continuously and strongly expressed at the apical side of single-layered cells in metaplastic gastroids cultured alone, but was only intermittently and faintly expressed in cells within polypoid projections in gastroids co-cultured with metaplasia- or cancer-derived fibroblasts (Supplementary Figure 11*B* and *D*). Co-cultured gastroid cells exclusively exhibited TROP2 and CEACAM5 expression, especially in protruding polyps, with preparations of fibroblasts from 3 different patients (Supplementary Figure 11*B* and *D*). In contrast with the effects of metaplasia- or cancer-derived fibroblasts on metaplastic cells, true normal-derived fibroblasts, isolated from histologically normal corpus mucosa without metaplasia or neoplasia obtained from the distal margins of esophagogastrectomies, did not promote polyp formation or expression of TROP2 or CEACAM5 compared with gastroid cells alone (Supplementary Figure 12*B–D*). These normal fibroblast populations were mainly composed of FbS3 and FbS4 (Supplementary Figure 12*A*).

### Conditioned Media From Metaplasia- or Cancer-Derived Fibroblasts Induce Dysplastic Transition in Metaplasia

**Figure 7:**
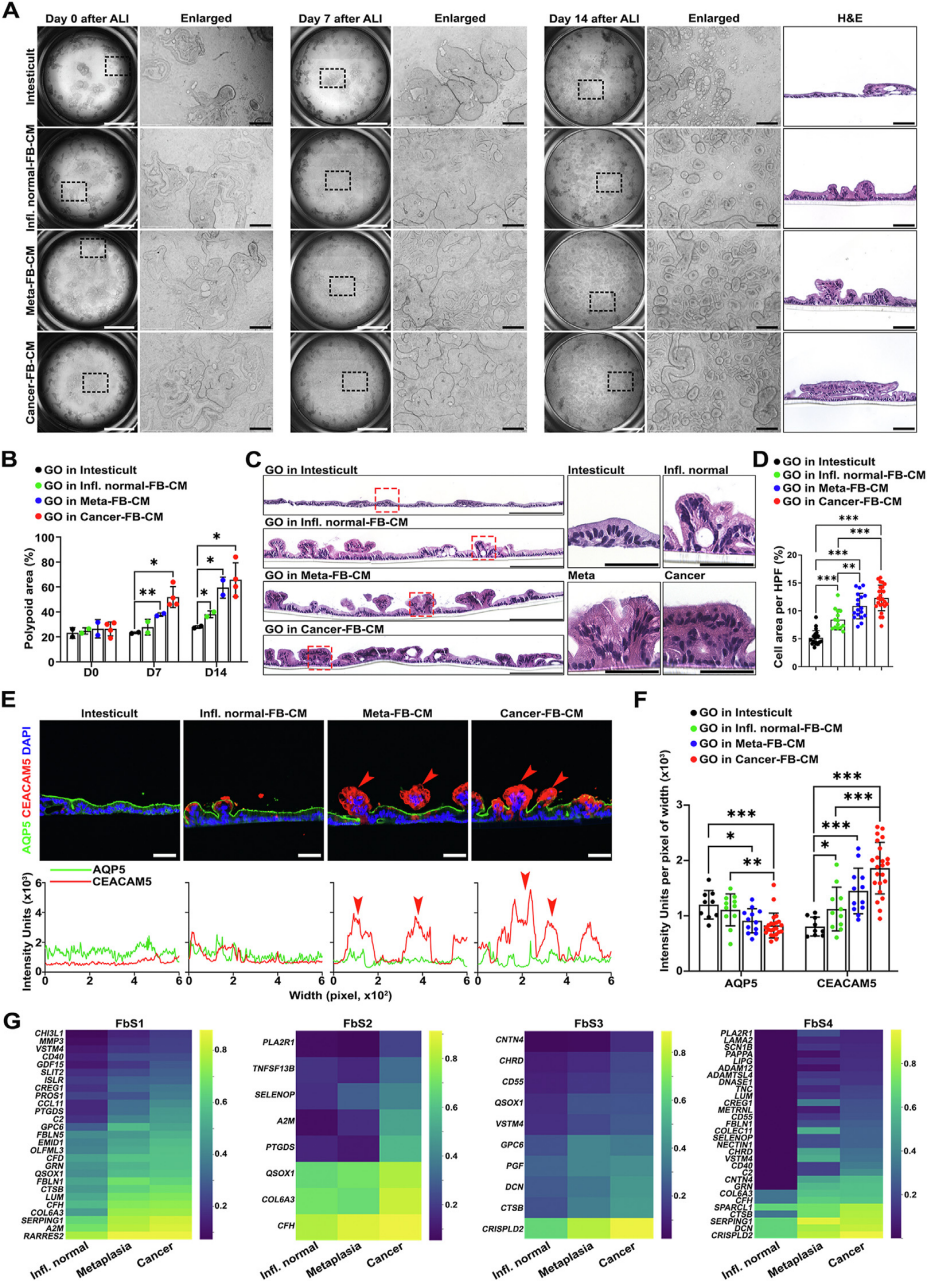
ALI culture with fibroblast (FB)-derived conditioned media (CM) demonstrates that secreted factors from FBs can induce dysplastic transition in metaplastic (Meta) cells. (*A*) Representative *brightfield images* of ALI co-culture of Meta gastroid cells in CM from inflamed normal- (Infl. normal-), Meta-, or cancer-derived FBs at each time point, all from patient 2. Paired *H&E images* show morphologic differences in polyp formation. *Scale bars:* 2000 μm and 200 μm for enlarged or 100 μm for H&E. (*B*) Quantification of the proportion of projected area from the base of filters shown in (*A*). Data are presented as mean ± SD (n = 2 independent experiments composed of CM from inflamed normal-, Met-, or cancer-derived FBs from patient 2 and cancer-derived FB from patient 1). ∗*P* < 0.05; ∗∗*P* < 0.01. (*C*) Representative H&E *images* of an entire filter of each ALI culture condition. *Red boxes* are enlarged on the *right side*, demonstrating morphologic alterations and different cell density in the polyps for each condition. *Scale bars:* 200 μm and 50 μm for enlarged. (*D*) Quantification of the area occupied with gastroid cells under high-power fields. Data are presented as mean ± SD (n = 2 independent experiments). ∗∗*P* < 0.01; ∗∗∗*P* < .001. (*E*) Representative *images* of immunofluorescence staining for metaplasia marker AQP5 and dysplasia marker CEACAM5 with nuclear 4′,6-diamidino-2-phenylindole (DAPI) and matched profiling of the expression of 2 markers. *Arrowheads* indicate polyps expressing CEACAM5. *Scale bars:* 50 μm. (*F*) Quantification of the staining shown in (*E*), presenting total expression levels for the 2 markers in each condition. Data are presented as mean ± SD (n = 2 independent experiments). ∗*P* < .05; ∗∗*P* < .01; ∗∗∗*P* < .001. (*G*) *Heatmaps* of up-regulated genes in each FB subset from metaplasia- or cancer-bearing tissues compared with the same subset from inflamed normal tissues, derived from the candidate genes described in Supplementary Figure 13*B–E*.

To investigate whether secreted factors from fibroblasts could promote dysplastic progression of metaplasia without direct contact, we cultured metaplastic gastroids in ALI conditions with regular media or with addition of conditioned media from inflamed normal-, metaplasia-, or cancer-derived fibroblasts. Although gastroid cells in regular media did not show any significant changes in morphology at day 7 after ALI, the polypoid area of gastroid cells grown in conditioned media expanded (Figure 7*A* and *B*). In addition, conditioned media from metaplasia- or cancer-derived fibroblasts not only promoted polyp formation in gastroid cells, but also increased cellularity in the polyps at day 14 after ALI, compared with regular or conditioned media from inflamed normal-derived fibroblasts (Figure 7*A–C*). Based on these structural changes, we next evaluated lineage marker expression for AQP5 or CEACAM5, which define metaplasia or dysplasia, respectively. Monolayered gastroid cells in regular media were consistently positive for AQP5, and negative for CEACAM5. However, the epithelial cells in polypoid structures observed in cultures with conditioned media from metaplasia- or cancer-derived fibroblasts strongly expressed CEACAM5 at the apical side with weak or negative AQP5 expression (Figure 7*D* and *E*). Although AQP5^+^ monolayered gastroid cells also expressed MUC6, a mucin observed in SPEM, CEACAM5^+^ cells in polyps were essentially negative for MUC6 or MUC2, a type of intestinal mucin, suggesting direct conversion from SPEM cells to dysplastic cells (Supplementary Figure 13*A*). We also examined the effect of fibroblast-derived conditioned media on epithelial proliferation. In contrast to the results from direct co-culture with fibroblasts, conditioned media even from cancer-derived fibroblasts did not promote gastroid cell proliferation (Supplementary Figure 13*A*).

We next sought to identify candidate secreted factors from fibroblasts that may be responsible for dysplastic progression. Among up-regulated genes in metaplasia- or cancer-derived fibroblasts compared with inflamed normal-derived fibroblasts (fold-change cutoff = 2, false discovery rate < 0.05), 79 or 96 candidate genes, respectively, were sorted from a database (http://genomics.cicbiogune.es/VerSeDa) as candidates encoding secreted molecules (Supplementary Table 2). Among them, 58 genes were observed in both groups.^30^ Construction of a protein*–*protein interaction network of the candidates revealed that most of the genes were functionally connected to each other. Furthermore, 2 networks from metaplasia- or cancer-derived fibroblasts shared several hubs (Supplementary Figure 13*B*). In patient-derived scRNA-seq, these candidate genes were more frequently expressed in FbS1 and FbS2, which were the main components of metaplasia- or cancer-derived fibroblasts (Supplementary Figure 13*C* and *D*). Notably, metaplasia or cancer tissue*–*derived FbSs showed higher expression levels of several candidate genes compared with the same subset from inflamed normal tissues (Figure 7*F*). Gene Ontology analysis demonstrated that the candidate genes were functionally enriched for “extracellular matrix,” “cell proliferation,” “cell adhesion,” or “secretion”-related pathways (Supplementary Figure 13*E*). These results imply that specific subsets of fibroblast secreted factors may account for distinct phenotypes at different pathologic stages.

## Discussion

Previous investigations in a number of systems have noted the importance of fibroblasts in the maintenance of both physiological and pathophysiological mucosae. In the present study, we identified 4 FbSs through scRNA-seq data analysis that were present in normal stomach and displayed distinctive geographical distributions with differential expansion in the context of metaplastic or dysplastic mucosae. Based on the prominent expression of PDGFRα, the close proximity to the epithelium and the morphologic features of extended cytoplasmic processes, FbS1 likely represents a telocyte-like population that can be distinguished from other fibroblasts, such as αSMA^+^ myofibroblasts.^31^^,^^32^ The PDGFRα^+^ telocytes in human and mouse intestine are located in the subepithelial regions, playing important roles in maintaining epithelial integrity, transduction of sensory signals, and serving as a component of the stem cell niche.^27^^,^^31^ PDGFRα^+^ telocytes in the pericryptal region of the colonic mucosa are a major source of several WNT ligands and BMP, which promote a balance between proliferation and differentiation of colonic epithelial cells.^33^ In accordance with these features, FbS1 cells expressing PDGFRα in adjacent normal stomach were mainly located in the isthmus region of the stomach corpus, where progenitor cells reside, which was distinct from the location of αSMA^+^ FbS3 myofibroblasts. In addition to the topography, FbS1 in single-cell transcriptomics uniquely expressed *WNT4*, *WNT5A*, *BMP2*, and *BMP4*, implicating FbS1 fibroblasts as the major functional telocyte population in stomach. Nevertheless, the levels of plasticity among fibroblast populations remain unclear.^12^

Previous studies have reported the association of fibroblasts, also called CAFs, with gastric cancer cell physiology, including promotion of proliferation, migration, and metastasis.^34^ A previous study using a bone marrow transplantation mouse model also found that *Helicobacter* infection*–*induced recruitment of bone marrow*–*derived mesenchymal stem cells can give rise to niche for gastric carcinogenesis.^35^ Recruitment of the cells to gastric mucosa is known to be dependent on CXCL12, CXCL16, or TGFβ secreted by epithelial or immune cells, and the recruited cells usually express high levels of soluble molecules, such as IL-6, WNT5A, BMP4, or CCL5.^36^ Nevertheless, the subsets of fibroblasts or CAFs and their functions during the gastric carcinogenic process had not been elucidated in detail. Kumar et al^17^ recently suggested that the presence of a *FAP*^*+*^ and *INHBA*^*+*^ CAF subset is associated with poor prognosis of gastric cancer through single-cell transcriptomic analysis. Our present studies indicate that the patterns of fibroblasts promoting early carcinogenesis in the stomach are quite different from previous reports in other organ systems. In contrast with CAFs associated with pancreatic ductal adenocarcinoma, myofibroblasts (FbS3) do not seem to be strongly associated with promotion of gastric carcinogenesis. Contrarily, telocyte-like fibroblasts (FbS1) appear to play a major role in establishing a metaplastic and dysplastic niche in gastric carcinogenesis, with expansion of the FbS1 population in close contact with metaplastic and dysplastic cell lineages at the bases of preneoplastic glands. These FbS1 cells are the most significant source of WNT ligands in the metaplastic and dysplastic stomach, and recent studies have demonstrated that dysplastic stem cell expansion and progression is driven by WNT activation.^37^ In addition, we also found that several factors facilitating cell growth and remodeling of extracellular matrix are enriched in FbS1 and FbS2 cells derived from metaplastic or cancer tissues. Thus, the gastric precancerous milieu may be driven by the combined action of telocyte-like FbS1 cells and inflammatory CAF-like FbS2 cells rather than myofibroblasts.

We have sought to identify markers that define the transitions between metaplasia and dysplasia, particularly for incomplete intestinal metaplasia, which represents the pathologic finding most associated with progression to gastric cancer.^5^ Previous studies have shown that AQP5 and CD44v9 are absent in the normal stomach corpus, but are up-regulated in SPEM lineage cells.^7^^,^^21^^,^^38^ AQP5 is positive in SPEM lineage cells at the bases of glands of both pyloric metaplasia and incomplete intestinal metaplasia,^7^ but AQP5 is down-regulated in dysplasia. TROP2 is up-regulated in intestinal lineage cells in incomplete intestinal metaplasia and dysplasia, but is absent from SPEM cells or complete intestinal metaplasia glands.^21^ Our present studies indicate that CEACAM5 is expressed in the intestinal lineage cells within incomplete intestinal metaplasia and more strongly in dysplasia. Previous studies have noted the up-regulation of CEACAM5 in gastric cancers.^24^ We have used these marker transitions to define the effects of fibroblasts on human metaplastic gastroids that predominantly display the characteristics of SPEM lineages, with prominent expression of CD44v9 and AQP5, but without significant expression of TROP2 or CEACAM5. In ALI Transwell cultures, the SPEM gastroids grew mostly as monolayers with prominent expression of CD44v9 and AQP5. When they were co-cultured with fibroblasts from true normal gastric mucosa, they maintained their monolayer growth phenotype with expression of CD44v9 and AQP5. However, co-culture of SPEM gastroids with fibroblasts derived from either metaplastic or cancer-bearing mucosae induced a profound change in growth pattern with development of multiple polypoid lesions with multiple lumens. Most importantly, the polypoid lesions showed a marked decrease in CD44v9 and AQP5 expression, while also demonstrating prominent increases in the expression of TROP2 and CEACAM5. Because we did not see evidence for intestinalization during the entire period of co-culture, these findings indicate that SPEM lineage cells under the influence of altered fibroblast populations may evolve to dysplasia without intermediate conversion to fully intestinalized cell lineages. Furthermore, SPEM gastroids under the influence of conditioned media from metaplastic- or cancer-derived fibroblasts recapitulated dysplastic transition with an increase in the dysplasia marker CEACAM5 and a concomitant loss of the metaplasia marker AQP5. However, an increase in proliferation was not induced with conditioned media in the gastroids. Thus, while some stable secreted factors and matrix components are likely present in the conditioned medium, fibroblasts likely secrete labile factors that can promote metaplasia to dysplasia transition and proliferation.^12^ In addition, gradients of factor concentration may be important, and thus apposition of fibroblasts adjacent to epithelial cells is likely important for the full manifestation of dysplastic progression.

In summary, we have performed a directed analysis of the precancerous milieu in patients with gastric cancer. Studies using scRNA-seq have established discrete subsets of fibroblasts in the normal and pathologic gastric mucosae that demonstrate distinct geographical patterns for association with normal, metaplastic, and dysplastic epithelial cell lineages. Furthermore, isolated fibroblasts from metaplastic and cancer-containing mucosae can directly promote progression of metaplastic SPEM gastroid cells to dysplasia. These findings demonstrate the critical role of distinct FbSs and their interaction with epithelial cells in promoting dysplastic transitions in the stomach.
