# Supplementary material for: Apposition of Fibroblasts With Metaplastic Gastric Cells Promotes Dysplastic Transition
Source: Gastroenterology. 2023 Aug;165(2):374–90. doi: 10.1053/j.gastro.2023.04.038 (PMC10375042; doi:10.1053/j.gastro.2023.04.038)
Supplement: Supplementary Materials [file mmc1.pdf]

Supplementary materials for

Apposition of fibroblasts with metaplastic gastric cells promotes dysplastic transition.

Supplementary Methods

Tissue acquisition and sample preparation

Fresh tissues were obtained from consented patients with treatment naive gastric adenocarcinoma who underwent gastrectomy at Montreal General Hospital- McGill University Health Centre, Montreal, Quebec, Canada (Research Ethics Board No. 2007-856). Tissues were collected from tumor and adjacent non-tumor regions which were suspected to contain intestinal metaplasia (usually within 1-2 cm proximal to the tumor) and normal corpus from the proximal region of the resection. Hematoxylin and eosin - stained sections of formalin-fixed paraffin-embedded (FFPE) tissue blocks taken from the same tissues used for gastroid and fibroblast preparations were used to corroborate the tissue pathology by consensus of two expert gastro-intestinal pathologists (Supplementary Figure 8A). Normal gastric mucosal fibroblast samples were derived from the distal gastric resection margins of two esophago-gastrectomies for esophageal adenocarcinoma. Hematoxylin and eosin staining of the gastric tissues from the distal margin showed normal gastric mucosa without metaplasia or cancer and lacking significant inflammation. Collected tissue specimens were divided in equal sections and placed in cold media (RPMI (Invitrogen)) containing Primocin (Invivogen) and gentamycin (Invitrogen)) for single cell RNA sequence processing or in Belzer UW®

Cold Storage Solution (Bridge to Life) containing 0.1% Y-27632 (Sigma) for gastroid establishment.

Single cell RNA sequencing

Single cell dissociation

Tissue was dissected to remove necrotic areas, minced and digested in 5 mL of Advanced DMEM/F12 containing 10 mg Collagenase Type 3 (Worthington) and 500 U Hyaluronidase (Sigma) in a C-tube (Miltenyi) using the gentleMACS Octo Dissociator (Miltenyi). The single cell suspension was resuspended in PBS and 1mM DTT, strained through a 100um cell strainer (Fisher) and spun down (500xg, 5 minutes, 4^oC). Cells were resuspended in 0.25% Trypsin-EDTA (Invitrogen) and incubated for 5 minutes at 37^oC, followed by addition of 10% fetal bovine serum to inactivate trypsin. The cell pellet (500xg, 5 minutes, 4^oC) was resuspended in 2.5U Dispase/10ug DNAse buffer and incubated for 5 minutes at 37^oC. The buffer was inactivated by adding excess PBS and the homogenate was strained (40 uM, Fisher) prior to centrifugation (500xg, 5 minutes, 4^oC). Red blood cells were lysed using ACK Lysing Buffer (Gibco) for 5 minutes at room temperature (RT), followed by addition of excess PBS, prior to centrifugation (500xg, 5 minutes, 4^oC). The cell pellet was finally washed twice with 2% fetal bovine serum in PBS prior to proceeding with single cell capture on the 10x Genomics platform.

Single cell suspension quality assessment

Prior to single cell capturing we assessed the single cell viability, the overall presence of debris and erythrocytes in our single cell suspension. Upon adequate

viability, lack of debris and erythrocytes the cells were captured on the 10x Genomics platform.

Before profiling the cell suspension, the cells were filtered through a 40 um FLOWMI cell strainer (SP Bel-Art; H13680-0040). Whenever necessary, centrifugation of the cells happened at 300 x g for 11 minutes.

Cell viability was tested using the "LIVE/DEAD Viability/Cytotoxicity Kit for mammalian cells" that contained the dyes Ethidium Homodimer-1 and Calcein-AM stain (ThermoFisher; L-3224). First, a viability stain mix was made which consisted of 0.5 ul of 4mM Calcein-AM, 2ul of 2mM Ethidium Homodimer-1 and 100ul of PBS. Then a 5ul of cell suspension was re-suspended in 5ul of viability stain mix and the solution was incubated at RT for up to 10 minutes. The sample viability was verified using a hemocytometer (INCYTO C-Chip; DHC-N01-5) through GFP (for the Calcein-AM) and RFP (for the Ethidium Homodimer-1) channels on an EVOS FL Auto Fluorescent microscope (ThermoFisher). Viability was expressed as the percentage of live cells (Calcein-AM / GFP positive cells) over the sum of live (Calcein-AM / GFP positive cells) and dead cells (Ethidium Homodimer-1 / RFP positive) cells.

The erythrocyte contamination was assessed by staining the cells with a cell permeable DNA dye DRAQ5 (ThermoFisher; 65-0880-92). A nuclear staining mix was made by diluting the DRAQ5 stock solution (5mM) down to 5uM with 1x PBS. Afterwards, a 5ul of cell suspension was re-suspended in 5ul of nuclear stain mix and the solution was incubated at RT for 5 minutes. The nuclear stain was verified using a hemocytometer (INCYTO C-Chip; DHC-N01-5) through the Cy5 channels on an EVOS FL Auto Fluorescent microscope (ThermoFisher). Erythrocyte contamination was

expressed as the percentage of “round donut-shaped DRAQ5 negative objects on bright-field” over the sum of “round donut-shaped DRAQ5 negative objects on bright-field” and “nuclear stained DRAQ5 positive cells”.

Additionally, we assessed the cell suspension for the presence of any other contaminants/debris as well as contaminants that might interfered with the capturing on the microfluidic chip for example large debris. The percentage of debris presented in the sample was expressed as follows: Percentage of “observed non-cell objects on bright-field” over the sum of “observed objects on bright-field” and “observed cells marked on the fluorescent channels”.

A sample was deemed adequate for capturing if “cell viability” >=70%, “erythrocyte contamination” <=10% and “debris percentage” <=30%.

To calculate the concentration of cells in the cell suspension we measured the number of “Calcein-AM / GFP positive cells” and “Ethidium Homodimer-1 / RFP positive cells” in the large 4 squares on each corner of the hemocytometer and the concentration of cells was calculated as follows: Number of cells / μl = [(“Calcein-AM / GFP positive cells” + “Ethidium Homodimer-1 / RFP positive cells”) / 4] * 10 * 2

where 10 was the dilution factor on the hematocytometer and 2 was the dilution factor when the cell suspension was mixed with the dye solution.

Single cell capturing

The single cells were captured on the 10x Genomics platform. For single cell 3’ end gene expression profiling we followed the “Chromium Next GEM Single Cell 3’ Reagent Kits v3.1” protocol and we used the corresponding reagents. For the single cell Copy

Number Variation, we followed the “Chromium Single Cell DNA Reagent Kits” protocol and we used the corresponding reagents. We note here that the presented CNV kit, as of currently, is discontinued. The sequencing libraries were created as per the above protocols with the modifications presented in the following section.

MGI and Illumina sequencing of the 10x single cell libraries

Libraries were quantified using a LightCycler 480 Real Time PCR instrument (Roche) and the KAPA library quantification kit (Roche) with triplicate measurements. Library quantification values were used both for the MGI library conversion and for Illumina sequencing normalization.

Libraries sequenced on MGI (MGI Tech) were converted after 10x library construction, to be compatible with MGI sequencers using the MGIEasy Universal Library Conversion Kit. The kit circularizes the libraries making them compatible for MGI systems. To sequence the circularized libraries, they were first amplified by rolling circle amplification, resulting in a long DNA strand which individually folds into a tight ball called a DNA nanoball where one library fragment results in one DNA nanoball. Before loading into the flowcells, the amplified nanoballs were quantified with a Qubit ssDNA HS Assay kit (ThermoFisher), normalized and loaded onto the sequencing flowcell using the auto-loader method (auto-loader MGI-DL-200R). The flowcells have a functionalized surface that captures and immobilizes the nanoballs in a grid pattern. Typically, two libraries were loaded per lane for the single cell RNA libraries. For single cell RNA libraries sequenced with MGI, kit DNBSEQ-G400RS

PE100 with App-A primers. For single cell DNA libraries, we used MGI kits DNBSEQ-G400RS PE150 with App-A primers.

The flowcells were sequenced on MGI sequencer model DNBSEQ-G400. For single cell RNA libraries, we sequenced 28 cycles for read1, 150 cycles for read2 and 8 cycles for the i7 index. For the single cell DNA libraries, we sequenced 151 cycles for Read1, 151 cycles for Read2 and 8 cycles for the i5 index. Is it to be noted that the libraries must be color balanced for all cycles sequenced as to maintain a minimum ratio of 0.125 for each base at each cycle, consequently color balanced single index adapters from 10x Genomics were used for libraries sequenced on MGI.

A subset of 23 libraries were sequenced on the Illumina NovaSeq 6000 platform using S4 flowcells. To ensure uniform loading of the libraries here, a preliminary pool was sequenced on Illumina iSeq and the library proportions were readjusted accordingly. Another subset of 12 libraries were sequenced on the Illumina HiSeq 4000 system typically with one library per lane.

The MGI sequencer has onboard capability to demultiplex samples, but we chose to use independent tools to demultiplex the raw fastq files for each lane to give us the flexibility to reprocess if needed. The fastqs generated using the balanced single index adapters also need to be merged for each library after demultiplexing, this is incorporated as an additional step after demultiplexing. The MGI runs were mainly demultiplexed by fastq-multx (https://github.com/brwnj/fastq-multx) but also using fgbio/DemuxFastqs (http://fulcrumgenomics.github.io/fgbio/tools/latest/DemuxFastqs.html). In both cases we used a mismatch of 1. Illumina runs were demultiplexed by the standard bcl2fastq tool.

Before downstream analysis, single cell RNA data were processed by cellranger-count version 3.0.1 (10x Genomics) and single cell DNA data were processed by cellranger-cnv version 1.1.0 (10x Genomics).

Bioinformatic methods

Read processing and alignment

After polyA-trimming via cutadapt (v3.2) [1], reads were pseudoaligned to the GRCh38 reference transcriptome (ENSEMBL release 96) with kallisto (v0.46.2) [2] using the default kmer size of 31. The pseudoaligned reads were processed into a cell-by-gene count matrix using bustools (0.40.0) [3]. Cell barcodes were filtered using the whitelist (v3) provided by 10xGenomics. All further processing was done in scanpy (v1.9.1) [4].

Quality control and normalization

Quality control was performed for each sample independently as follows: Cell barcodes with less than 1000 counts or less than 500 genes expressed were removed. Cell barcodes with more than 20% mitochondrial gene expression were removed. Doublet cells were identified using scrublet [5], removing any cell barcode with a scrublet score > 0.2. Only coding genes were retained in the final count matrix. Expression profiles were normalized by total counts, the 4000 most highly variable genes identified [6], renormalized, log-transformed and z-scored. The data was projected onto the first 50 principal components.

Data integration, visualization, clustering

After the above "per-sample" preprocessing, samples are pooled and integrated using Harmony [7] on the first 50 principal components with a maximum of 25 iterations. A nearest neighbor graph (k=15) was calculated on the harmony corrected principal components space. Datasets were visualized in 2D via UMAP [8], initialized with PAGA [9] coordinates. The nearest neighbor graph was clustered with the Leiden algorithm [10]. The datasets were visualized interactively using Cell Browsers [11] allowing for easy access and exploration across teams and labs. Cluster markers were determined using Wilcoxon tests with Benjamini-Hochberg p-value adjustment; within clusters, genes were ranked by statistic.

Plots were produced using python libraries seaborn and scanpy. For Figure 7*G*, the proportion of cells expressing a given gene (y axis) from the secreted list in Supplementary Table 2 is shown. The genes are subset from the secreted list so that the (% cells expressing in Cancer) - (% cells expressing in Inflamed normal) is greater than 10%, and sorted by the proportion of cells expressing in Cancer. In Supplementary Figure 4*C*, fractions of cells in dotplots were based on a cell having greater than 5 counts, except in Figure 3*B* where the threshold was lowered to greater than 1 count due to important marker genes being expressed at a very low rate. For Supplementary Figure 13*D*, the list of 114 secreted genes (intersected from Supplementary Table 2) was used to subtype a matrix of gene expression for the 2319 fibroblasts. Then, each gene was normalized across the cells. The normalized matrix of expression was summed for each gene. The cells were summarized as boxplots

grouped by fibroblast subtype so that the sum of each gene across cells is controlled, and each cell has the same potential expression relative to other cells.

Cell type calling

Initial cell types were called using the Human Cell Landscape reference dataset [12]. Briefly, the raw expression profile x_i of cell i was normalized by total counts and log-transformed: $\mathrm{y_{ij}} = \log\left(1 + \mathrm{x_{ij}} / \sum_{\mathrm{j}} \mathrm{x_{ij}}\right)$ with $\mathrm{x_{ij}}$ the counts of gene j in cell i. Cells were compared to the Human Cell Landscape reference by Pearson correlation, and the reference profile with the highest correlation determined the cell type call. If the highest correlation was below 0.3, the cell type was defined as "Unknown". Later cell type calling was refined manually.

Bulk RNA-seq analysis

Quality control

Raw data (raw reads) of FASTQ format were firstly processed through fastp. In this step, clean data (clean reads) were obtained by removing reads containing adapter and poly-N sequences and reads with low quality from raw data. At the same time, Q20, Q30 and GC content of the clean data were calculated. All the downstream analyses were based on the clean data with high quality.

Mapping to reference genome

Reference genome and gene model annotation files were downloaded from genome website browser (NCBI/UCSC/Ensembl) directly. Paired-end clean reads were aligned to the reference genome using the Spliced Transcripts Alignment to a

Reference (STAR) software, which is based on a previously undescribed RNA-seq alignment algorithm that uses sequential maximum mappable seed search in uncompressed suffix arrays followed by seed clustering and stitching procedure. STAR exhibits better alignment precision and sensitivity than other RNA-seq aligners for both experimental and simulated data.

Quantification

FeatureCounts was used to count the read numbers mapped of each gene. And then RPKM of each gene was calculated based on the length of the gene and reads count mapped to this gene. RPKM, Reads Per Kilobase of exon model per Million mapped reads, considers the effect of sequencing depth and gene length for the reads count at the same time, and is currently the most commonly used method for estimating gene expression levels.

Differential expression analysis

Differential expression analysis between two conditions/groups (three biological replicates per condition) was performed using DESeq2 R package. DESeq2 provides statistical routines for determining differential expression in digital gene expression data using a model based on the negative binomial distribution. The resulting P values were adjusted using the Benjamini and Hochberg's approach for controlling the False Discovery Rate (FDR). Genes with an adjusted P value < 0.05 found by DESeq2 were assigned as differentially expressed.

Enrichment analysis

Gene Ontology enrichment analysis of differentially expressed genes was implemented by the clusterProfiler R package. Gene Ontology terms with adjusted P value less than 0.05 were considered significantly enriched by differential expressed genes. Web-based ShinyGO (version 0.77; http://bioinformatics.sdstate.edu/go/) was also used for visualization of enriched pathway analysis.

Isolation and maintenance of human gastroids and fibroblasts

Fresh tissues in the range of 300 – 1,300 mg were obtained from cancer and adjacent non-cancerous lesions and stored in Belzer UW® Cold Storage Solution (Bridge to Life) containing 0.1% Y-27632 (Sigma) at 4°C for 24 hours upon delivery from Canada to USA. Tissues were washed with ice-cold PBS containing 100 μg/mL of Primocin and cut into two pieces using a razor blade. Each tissue piece was fixed in 10% neutral buffered formalin (NBF) for FFPE preservation or used for the generation of gastroid and fibroblast lines. Stomach mucosa was separated from serosa along the muscle layer using a cell scraper and minced using a tissue chopper. For the gastroid generation, the mucosa was carefully transferred to pre-warmed digestion buffer (DMEM/F12 + 5% Fetal Bovine Serum (FBS) + 1 mg/mL collagenase type 1a + 50 μg/ml DNAse I) at 37°C and incubated for 30 minutes with gently shaking the plate at 220 rpm. After the digestion, pre-warmed quenching buffer (DMEM/F12 + 1% FBS + 0.1% Y-27632 + 1 mM DTT) was added, followed by centrifugation at 300 g for 5 minutes. Pellets were then washed in pre-warmed quenching buffer, strained through a 100 μm cell strainer and centrifuged. Finally, the glands-containing pellets were

resuspended in ice-cold Matrigel (Corning) and a drop of 30 μL Matrigel-gland mixture was plated in a 48-well plate. After the Matrigel drop was polymerized at 37°C for 30 minutes, Human IntestiCult media (StemCell Technology) supplemented with 1% of penicillin/streptomycin (Corning), 0.2% of MycoZap (Lonza) and 0.1% Y-27632 was added. The media was changed every 3 days. For the fibroblast isolation, a 6-well plate was scored in a grid pattern with a razor blade under sterile conditions. The minced mucosa was moved over the grid using a sterilized forceps. The plate was incubated at 37°C for 30 minutes and Fibroblast Growth Medium-2 (FGM-2, Lonza) was added to each well. After the fibroblasts crawled out from the anchored mucosa, each well was washed with pre-warmed PBS and the fibroblasts were split and maintained with fresh FGM-2 media in a humidified incubator at 37°C and 5% CO_2. The media was replaced every 2-3 days and the fibroblasts were split when the cells reached 70-80% confluency. Harvested fibroblast culture media were kept frozen at -80 C for Air Liquid Interface cultures with conditioned media.

Air Liquid Interace (ALI) cultures with fibroblast-derived conditioned media

Air Liquid Interface (ALI) cultures with conditioned media (CM) from various fibroblasts were performed with an identical method used in ALI co-culture. Briefly, gastroids in ~200 μl of Human IntestiCult Medium (StemCell Technology) were seeded onto the top of a collagen-coated Transwell filter, and 300 μl of Human IntestiCult Medium were added to the well under the Transwell filter. Top and bottom IntestiCult medium were changed after 48 hours. Seeded gastroid cells were left for 7 days to grow on top of collagen-coated Transwells. On day 7, the media overlying the cells was

removed to expose the surface of the cells to air, and the media under the Transwell filters was replaced with Human IntestiCult medium for control or with 1/3 conditioned media from the cultures of fibroblasts derived from inflamed normal-, metaplasia- or cancer-bearing mucosae, and then the ALI process lasted 14 days. The medium at the bottom wells was replaced every two days, and fluid secreted from the cells was removed every day from the top Transwells. After 14 days of ALI culture, Transwell filters from each ALI condition were washed in the plate with warmed PBS for 5 minutes and then fixed with 4% paraformaldehyde (PFA) for 20 minutes at RT. After fixation, the filters were washed with PBS for 5 minutes and then divided into two pieces for cryosection or paraffin embedding.

Fluorescence-activated cell sorting

For flow cytometry analysis, 5 x 10^5 of either true normal-, adjacent normal-, metaplasia- or cancer-derived fibroblasts were sorted with anti-PDPN, anti-CD248, anti-CD146, and anti-PDGFRβ antibodies based on the gating strategy as shown in Supplementary Figure S7. Briefly, harvested fibroblasts were washed with PBS containing 2% FBS twice and centrifugated at 1500 rpm, 4°C, followed by filtration using polypropylene round-bottom tubes with 100 μm cell strainer cap (BD Biosciences). Filtered cells were incubated with the antibody mixture with specific concentrations as indicated in Supplementary Table 3 for 30 minutes at 4°C. After washing with PBS containing 2% FBS once and centrifugation, the cells were briefly stained with DAPI in PBS containing 2% FBS for excluding dead cells. Flow cytometry was performed using 4-laser Fortessa (BD Biosciences) in the Vanderbilt Flow Cytometry Shared Resource.

Cryosection preparation and immunostaining

After fixation and washing, half of the filters from ALI cultures were processed under an infiltration step using 30% sucrose overnight at 4°C. Next day, the filters were cut out from inserts and then cut in half. For each insert, both halves were orthogonally embedded in OCT inside a casting mold and stored at -80°C before sectioning. Eight-micrometer sections were cut using a cryotome and placed on positively charged slides, the slides can be stored at -20°C before staining. Slides were let rest at RT for 20 minutes before staining. Then, they were fixed with cold acetone for 30 seconds and incubated in PBS for 10 minutes before staining to remove OCT. Sections were permeabilized and blocked with 0.1% Triton X-100, 10% NDS in PBS for 1 hour at RT. Slides were then washed for 5 minutes in PBS. Primary antibodies were diluted in 0.1% Triton X-100, 1% normal donkey serum (NDS) in PBS and incubated for 2 hours at RT. After washing 3 times in PBS for 5 minutes each, the slides were incubated secondary antibodies along with fluorescently-labeled Phalloidin for 2 hours in at RT. Hoechst diluted 1:5000 in PBS was added to each section for 5 minutes for counterstaining. Slides were washed 3 times for 5 minutes in PBS and mounted. Imaging was performed on a Nikon A1R or Zeiss LSM980 confocal microscope.

Human Gastric Cancer Microarrays

Two human gastric cancer microarray sets were constructed with specimens from 28 gastric cancer patients who underwent surgical resection in Jeju National University Hospital from 2014 to 2016 in Korea. Through histologic examination, cancer areas

were selected from H&E-stained slides. The tissue cores (4 mm in diameter) were obtained from each individual paraffin blocks and arranged in a recipient paraffin block using a trephine apparatus (SuperBioChips Laboratories, Seoul, Korea). Likewise, two human gastric corpus microarray sets were additionally generated from 18 gastric cancer patients who had surgical resection from 2015 to 2019. Tissue microarray construction was approved by the Institutional Review Board of Jeju National University Hospital (2016-10-001, 2022-01-009), and informed consent was waived by the Institutional Review Board due to the retrospective nature of the study.

Immunostaining

Five µm FFPE sections of tissue, gastroid or filter containing gastroid cells cultured under ALI conditionswere de-paraffinized in Histoclear and rehydrated through a series of ethanol (100%, 95%, 90%, 80% and 75%). The tissue sections were subjected to antigen retrieval with pH 6, Dako target retrieval solution in a pressure cooker for 15 minutes. Tissue sections were incubated in Dako Serum-free Protein Block Solution at RT for 90 minutes, and primary antibodies were applied overnight at 4°C (Supplementary Table 4). Next day, after washing in PBS for 5 minutes three times, the sections were incubated with secondary antibodies at RT for 60 minutes. Counterstaining was performed using diamidino-2-phenylindol (DAPI) in PBS for 5 minutes at RT. All immunofluorescence images of tissue sections with single case were captured with a Zeiss Axio Imager 2 using a 20X objective.

Whole mount staining

Matrigel domes containing co-cultured gastroids with fibroblasts in an 8-well chamber slide were washed with PBS three times and then fixed in 4% PFA. Non-specific binding was blocked with 250 μl of 0.3% Triton X-100 and 10% NDS in PBS per each well for 60 minutes at RT. After washing twice, primary antibodies in PBS containing 0.1% Tween 20 (PBST) and 1% NDS are applied overnight at 4°C. The following day samples were washed with PBST four times and then incubated with secondary antibodies in PBST with 1% NDS for 4 hours at RT. Counterstaining was performed using DAPI in PBS for 5 minutes at RT. Imaging for whole mount staining was performed on Nikon A1R confocal microscope.

Multiplexed immunofluorescence

For multiplexed immunofluorescence (MxIF) on tissue microarray slides, consecutive staining, imaging, and dye inactivation was implemented as described previously.[13] MxIF imaging was performed on Leica Aperio Versa 200 Fluorescent Slide Scanner in the VUMC Digital Histology Shared Resource (DHSR). Images were acquired at 20X magnification, and exposure times were determined on a per-antibody basis. Antibodies are listed in Supplementary Table 4, and antibody conjugations were performed with Zenon kit (Thermo) according to manufacturer's instructions.

Immunocytochemistry

Fibroblasts were seeded and maintained on the collagen-coated coverslips in a 12-well plate for 3 days. After removal of FBM, the plate was rinsed twice for 1 minute with PBS, and then the cells were fixed in 4% PFA for 20 minutes at RT. Permeabilization

was performed with 0.3% Triton X-100 in PBS for 20 minutes at RT. After washing twice with PBS, 10% NDS in PBST was applied for blocking non-specific binding for 1 hour at RT. Blocked samples were sequentially incubated with primary and secondary antibodies in PBS containing 1% NDS for 1 hour at RT per each step. After washing twice with PBST for 5 minutes each, the cells were counterstained with DAPI briefly and mounted on slides. Imaging was performed on Zeiss Axio Imager 2 using a 20X objective.

Toluidine Sections

Transwells were excised and fixed in 0.1M cacodylate buffer containing 2.5% glutaraldehyde at RT for 1 hour followed by overnight fixation at 4°C. Samples were washed and sequentially incubated with 1% tannic acid, 1% osmium tetroxide, and 1% uranyl acetate for 1 hour, followed by a graded ethanol dehydration. After dehydration, the samples were infiltrated with Spurrs resin and Quetol 651 using propylene oxide as a transition solvent, the resin was polymerized at 60°C for 48 hours. Thick sections (500 nm) were cut using a Leica UC7 ultramicrotome and collected onto glass-slides and stained with toluidine blue for 30s on hotplate. The thick sections were then mounted with coverslips using Spurrs resin as a mounting media and subsequently polymerized at 60°C. Imaging was performed on Zeiss LSM980 confocal microscope.

Image analysis

Three to four representative images were obtained from each stained section, and then positivity of each marker was manually or automatically quantitated using Cell

Profiler. Distance between epithelial membrane marked by pan-cytokeratin and the nucleus of neighboring fibroblast was manually measured with ZEN 3.2 software (Zeiss). Profiling of CEACAM5 and AQP5 expression levels along with width of each image was automatically performed with ZEN 3.2 software.

For analysis of multiplexed immunofluorescence (MxIF) staining, MxIF whole-slide image (WSI) extraction, registration, and layering were performed autonomously in MATLAB (The MathWorks, Inc.) with in-house built pipelines (DHSR) that rely on the Image Processing toolbox (The MathWorks, Inc.). Since subject slides were tissue microarrays, individual core tissue segments were automatically identified and extracted from the registered WSIs and successively re-registered. Captured bleach rounds of images were used to remove autofluorescence and background signals on a per wavelength/per marker basis. Positive signal was automatically determined by median in-tissue pixel intensities, also on a per marker basis. Subtracted images were loaded into ilastik 1.3.2 [14] to utilize machine learning to segment nuclear, membranous, cytoplasmic, etc. compartments. In brief, subregions of these core images were flipped and rotated as training material. Subregion types were annotated by Dr. Roland. Probability maps for each compartment were generated for each core image stack. Individual cells and their substructures were identified within each core image through its matched probability maps utilizing an automated pipeline (MATLAB). Individual marker intensity values were collected for each identified cell and were used as the determinates of cell classification. Euclidean distance mapping was performed within this pipeline, and the nearest neighbor distance of each target cell class was recorded. All statistical analyses were generated using unpaired two-tailed Student’s t test or

Repeated Measurements One-Way ANOVA Test with intra-group comparisons in GraphPad Prism 9 software (La Jolla, CA).

References

1. Martin M. Cutadapt removes adapter sequences from high-throughput sequencing reads. EMBnet. journal 2011;17:10-12.
2. Bray NL, Pimentel H, Melsted P, et al. Near-optimal probabilistic RNA-seq quantification. Nat Biotechnol 2016;34:525-7.
3. Melsted P, Booeshaghi AS, Liu L, et al. Modular, efficient and constant-memory single-cell RNA-seq preprocessing. Nat Biotechnol 2021;39:813-818.
4. Wolf FA, Angerer P, Theis FJ. SCANPY: large-scale single-cell gene expression data analysis. Genome Biol 2018;19:15.
5. Wolock SL, Lopez R, Klein AM. Scrublet: Computational Identification of Cell Doublets in Single-Cell Transcriptomic Data. Cell Syst 2019;8:281-291 e9.
6. Zheng GX, Terry JM, Belgrader P, et al. Massively parallel digital transcriptional profiling of single cells. Nat Commun 2017;8:14049.
7. Korsunsky I, Millard N, Fan J, et al. Fast, sensitive and accurate integration of single-cell data with Harmony. Nat Methods 2019;16:1289-1296.
8. Becht E, McInnes L, Healy J, et al. Dimensionality reduction for visualizing single-cell data using UMAP. Nat Biotechnol 2018;37:38–44.
9. Wolf FA, Hamey FK, Plass M, et al. PAGA: graph abstraction reconciles clustering with trajectory inference through a topology preserving map of single cells. Genome Biol 2019;20:59.
10. Traag VA, Waltman L, van Eck NJ. From Louvain to Leiden: guaranteeing well-connected communities. Sci Rep 2019;9:5233.
11. Speir ML, Bhaduri A, Markov NS, et al. UCSC Cell Browser: Visualize Your Single-Cell Data. Bioinformatics 2021;37:4578-4580.
12. Han X, Zhou Z, Fei L, et al. Construction of a human cell landscape at single-cell level. Nature 2020;581:303-309.
13. Gerdes MJ, Sevinsky CJ, Sood A, et al. Highly multiplexed single-cell analysis of formalin-fixed, paraffin-embedded cancer tissue. Proc Natl Acad Sci U S A 2013;110:11982-7.
14. Berg S, Kutra D, Kroeger T, et al. ilastik: interactive machine learning for (bio)image analysis. Nat Methods 2019;16:1226-1232.

Supplementary Figures

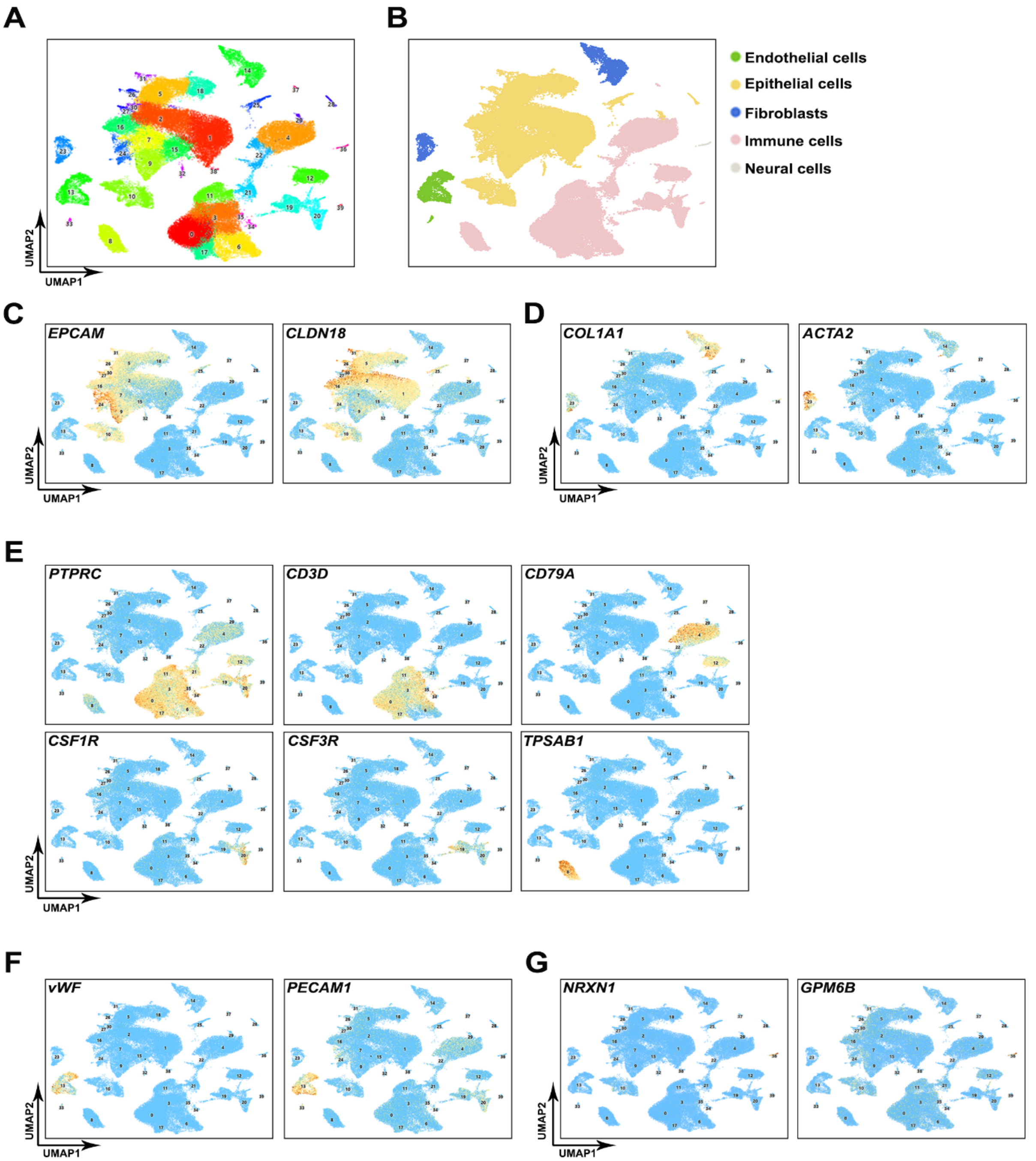

Supplementary Figure 1. Freshly collected 72,126 cells include all lineages that can be observed in stomach tissue of gastric cancer patients. A, Uniform Manifold

Approximation and Projection (UMAP) of whole cells in 39 color-coded clusters. Each dot in the UMAP indicates an individual cell. **B-G**, Re-clustered UMAP representing cell lineages marked with distinctive colors (B), divided into epithelial cells marked by *EPCAM* and *CLDN18* (C), fibroblasts marked by *COL1A1* and *ACTA2* (D), total immune cells marked by *PTPRC*, T cells marked by *CD3D*, B cells marked by *CD79A*, macrophages marked by *CSF1R*, neutrophils marked by *CSF3R* and mast cells marked by *TPSAB1* (E), endothelial cells marked by *vWF* and *PECAM1* (F) and neural cells marked by *NRXN1* and *GPM6B* (G).

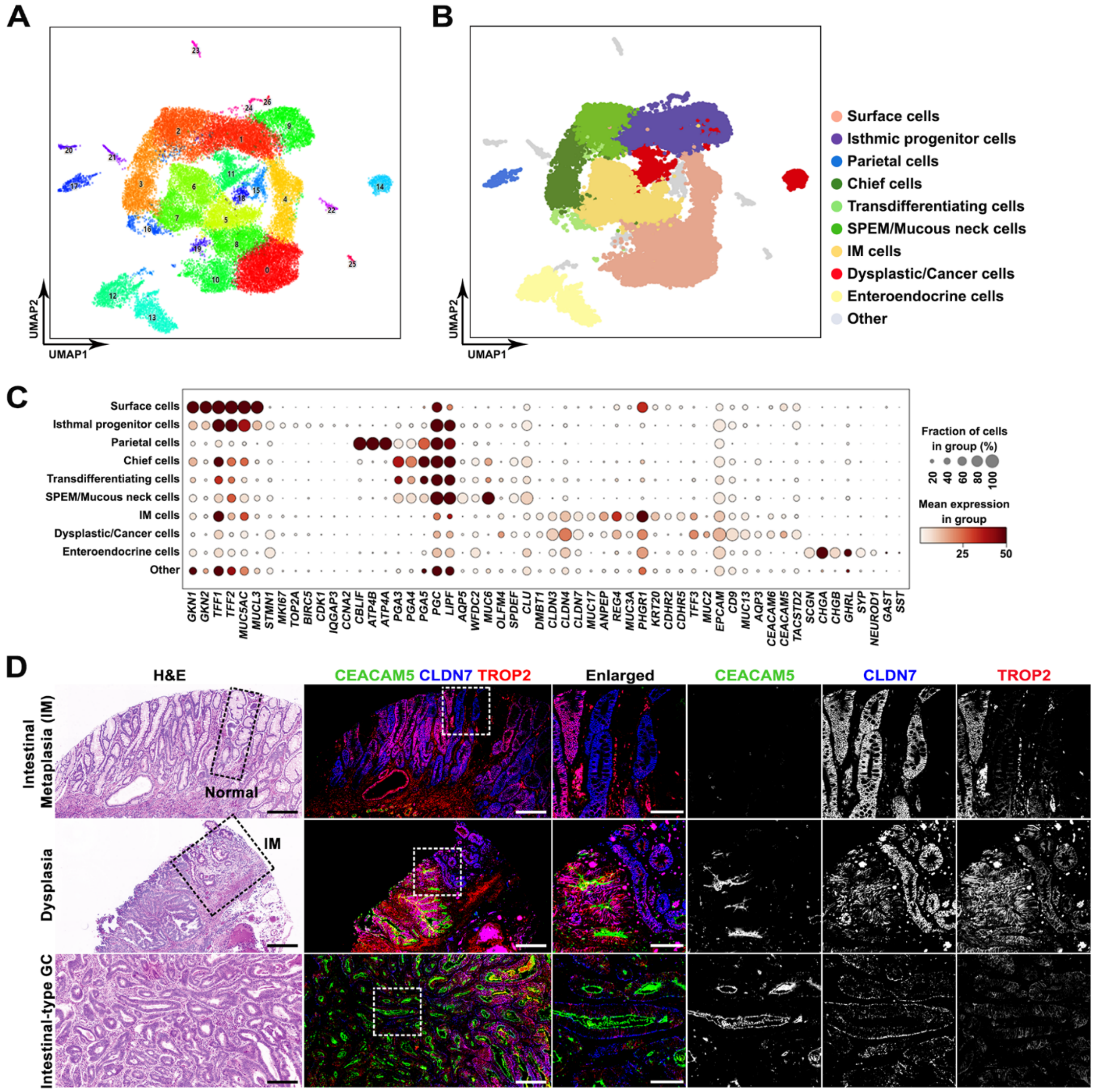

E

Pathology (Total ROI#)	CEACAM5	CLDN7	TROP2
Normal (5)	0/5	0/5	0/5
Incomplete IM (7)	2/7 (28.6%)	6/7 (85.7%)	5/7 (71.4%)
Dysplasia (11)	10/11 (90.9%)	7/11 (63.6%)	6/11 (54.5%)
Intestinal-type GC (13)	10/13 (76.9%)	7/11 (63.6%)	7/13 (53.8%)

Supplementary Figure 2. **scRNA-seq reveals molecular heterogeneity of epithelial cells in gastric carcinogenesis. A**, UMAP of 32,174 epithelial cells in 26 color-coded clusters. Each dot in the UMAP indicates an individual cell. **B**, UMAP color-coded according to cell-specific lineages of all epithelial cells in **A**. **C**, Bubble plot of normalized

expression of selected lineage-specific marker genes in epithelial cells belonging to a specific lineage described in **B**. **D**, Representative images of Hematoxylin & eosin (H&E) and immunofluorescence staining for potential dysplasia markers, CEACAM5, CLDN7 and TROP2, in tissue-microarray slide composed of human IM, dysplasia and intestinal-type gastric cancer. Each core was divided into several regions of interest based on histopathology. Scale bar=300μm and 100μm for enlarged. **E**, Summary table with the frequency of the expression of the potential dysplastic markers in different pathologic conditions.

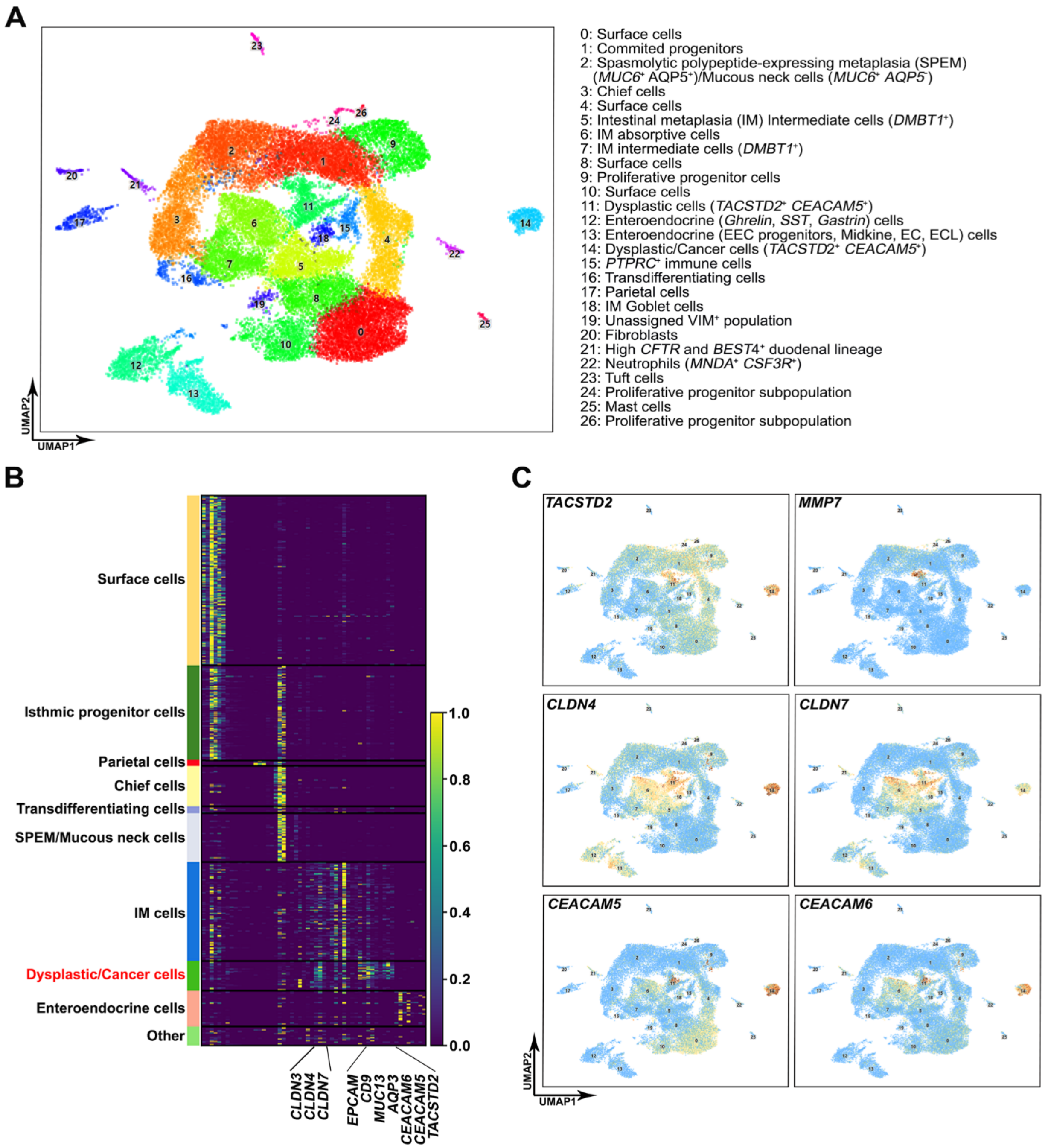

Supplementary Figure 3. scRNA-seq demonstrates cellular and molecular heterogeneity of epithelial cells in stomach tissues from gastric cancer patients and suggests several potential markers for dysplasia. A, UMAP representation of

32,174 epithelial cells in 26 color-coded clusters, as indexed on the right side of the UMAP. Each dot in the UMAP indicates an individual cell. **B**, Heatmap of selected genes enriched in scRNA-seq data of epithelial clusters, distinguished by the cell lineage. Rows indicate single cells. **C**, UMAPs representing expression of selected genes annotated in the heatmap of **B**, indicated by genes specific to each cluster.

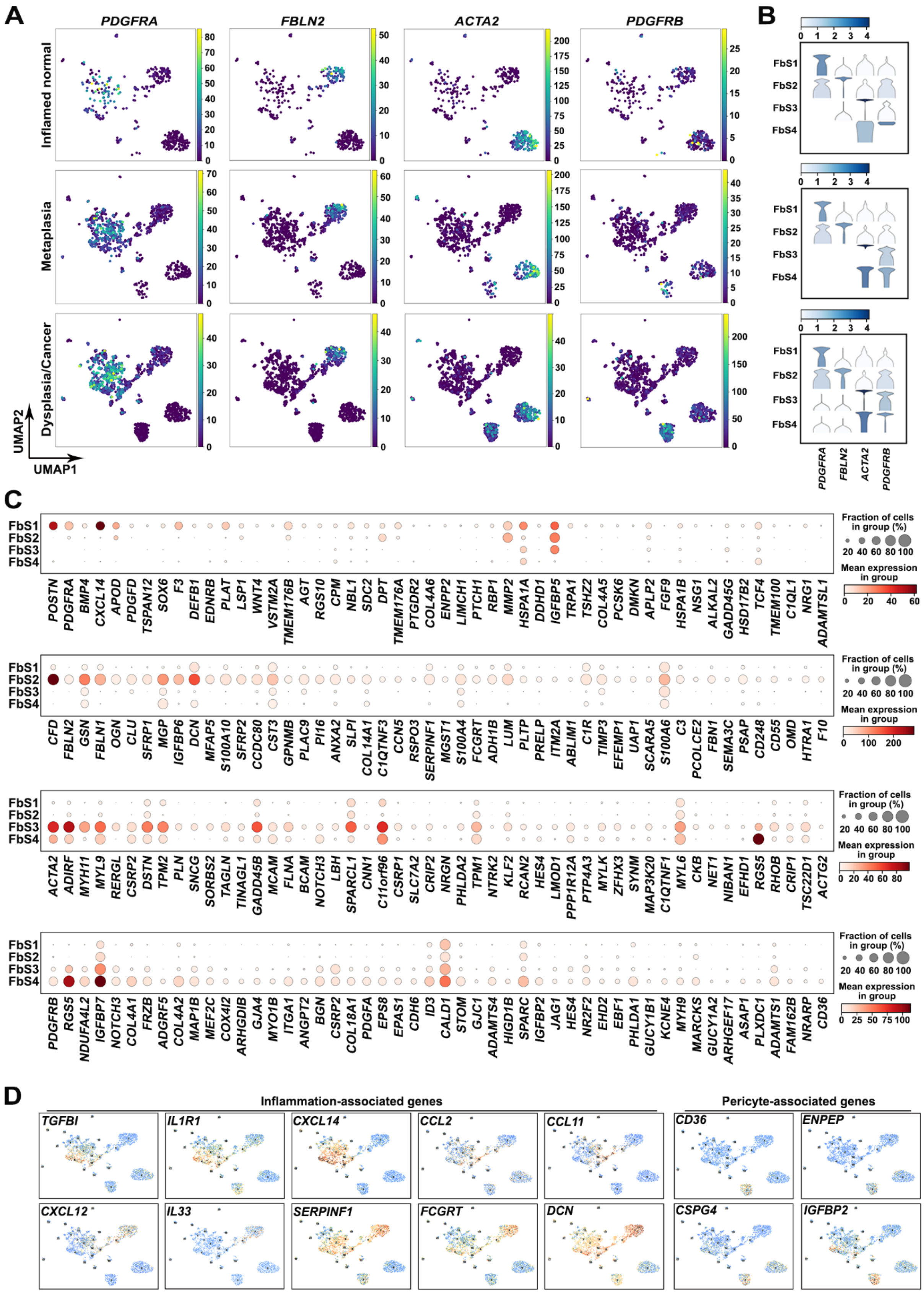
A
PDGFRA
FBLN2
ACTA2
PDGFRB
Inflamed normal
Metaplasia
Dysplasia/Cancer
UMAP2
UMAP1
B
FbS1
FbS2
FbS3
FbS4
PDGFRA
FBLN2
ACTA2
PDGFRB
C
FbS1
FbS2
FbS3
FbS4
POSTN PDGFRA BMP4 CXCL14 APOD PDGFD TSPAN12 SOX6 F3 DEFB1 EDNRB PLAT LSP1 WNT4 VSTM2A TMEM176B AGT RGS10 CPM NBL1 SDC2 DPT TMEM176A PTGDR2 COL4A6 ENPP2 LIMCH1 PTCH1 RBP1 MMP2 HSPA1A DDHD1 IGFBP5 TRPA1 TSHZ2 COL4A5 PCSK6 DMKN APLP2 FGF9 HSPA1B NSG1 ALKAL2 GADD45G HSD17B2 TCF4 TMEM100 C1QL1 NRG1 ADAMTSL1
CFD FBLN2 GSN FBLN1 OGN CLU SFRP1 MGP IGFBP6 DCN MFAP5 S100A10 SFRP2 CCDC80 CST3 GPNMB PLAC9 PI16 ANXA2 SLPI COL14A1 C1QTNF3 CCN5 RSPO3 SERPINF1 MGST1 S100A4 FCGRT ADH1B LUM PLTP PRELP ITM2A ABLIM1 C1R TIMP3 EFEMP1 UAP1 SCARA5 S100A6 C3 PCOLCE2 FBN1 SEMA3C PSAP CD248 CD55 OMD HTRA1 F10
ACTA2 ADIRF MYH11 MYL9 RERGL CSRP2 DSTN TPM2 PLN SNCG SORBS2 TAGLN TINAGL1 GADD45B MCAM FLNA BCAM NOTCH3 LBH SPARCL1 CNN1 C11orf96 CSRP1 SLC7A2 CRIP2 NRGN PHLDA2 TPM1 NTRK2 KLF2 RCAN2 HES4 LMOD1 PPP1R12A PTP4A3 MYLK ZFHX3 SYNM MAP3K20 C1QTNF1 MYL6 CKB NET1 NIBAN1 EFHD1 RGS5 RHOB CRIP1 TSC22D1 ACTG2
PDGFRB RGS5 NDUFA4L2 IGFBP7 NOTCH3 COL4A1 FRZB ADGRF5 COL4A2 MAP1B MEF2C COX4I2 ARHGDIB GJA4 MYO1B ITGA1 ANGPT2 BGN CSRP2 COL18A1 PDGFA EPS8 EPAS1 CDH6 ID3 CALD1 STOM GJC1 ADAMTS4 HIGD1B SPARC IGFBP2 JAG1 HES4 NR2F2 EHD2 EBF1 PHLDA1 GUCY1B1 KCNE4 MYH9 MARCKS GUCY1A2 ARHGEF17 ASAP1 PLXDC1 ADAMTS1 FAM162B NRARP CD36
Fraction of cells in group (%)
20 40 60 80 100
Mean expression in group
D
Inflammation-associated genes
Pericyte-associated genes
TGFBI IL1R1 CXCL14 CCL2 CCL11 CD36 ENPEP
CXCL12 IL33 SERPINF1 FCGRT DCN CSPG4 IGFBP2

Supplementary Figure 4. **Each gastric fibroblast subset has a distinctive gene signature.** **A**, UMAPs representing expression of selected markers for fibroblast or myofibroblast in each pathologic condition; inflamed normal, metaplasia and dysplasia/cancer. **B**, Violin plot of normalized expression of *PDGFRA*, *FBLN2*, *ACTA2* and *PDGFRB* in the different subsets from inflamed normal (top), metaplasia (middle) and dysplasia/cancer (bottom). **C**, Bubble plots representing normalized expression of top 50 differentially expressed genes (DEGs) in each fibroblast subset. **D**, UMAPs representing expression of selected genes related to inflammatory CAFs subpopulations and pericytes, indicated by genes specific to each fibroblast subset.

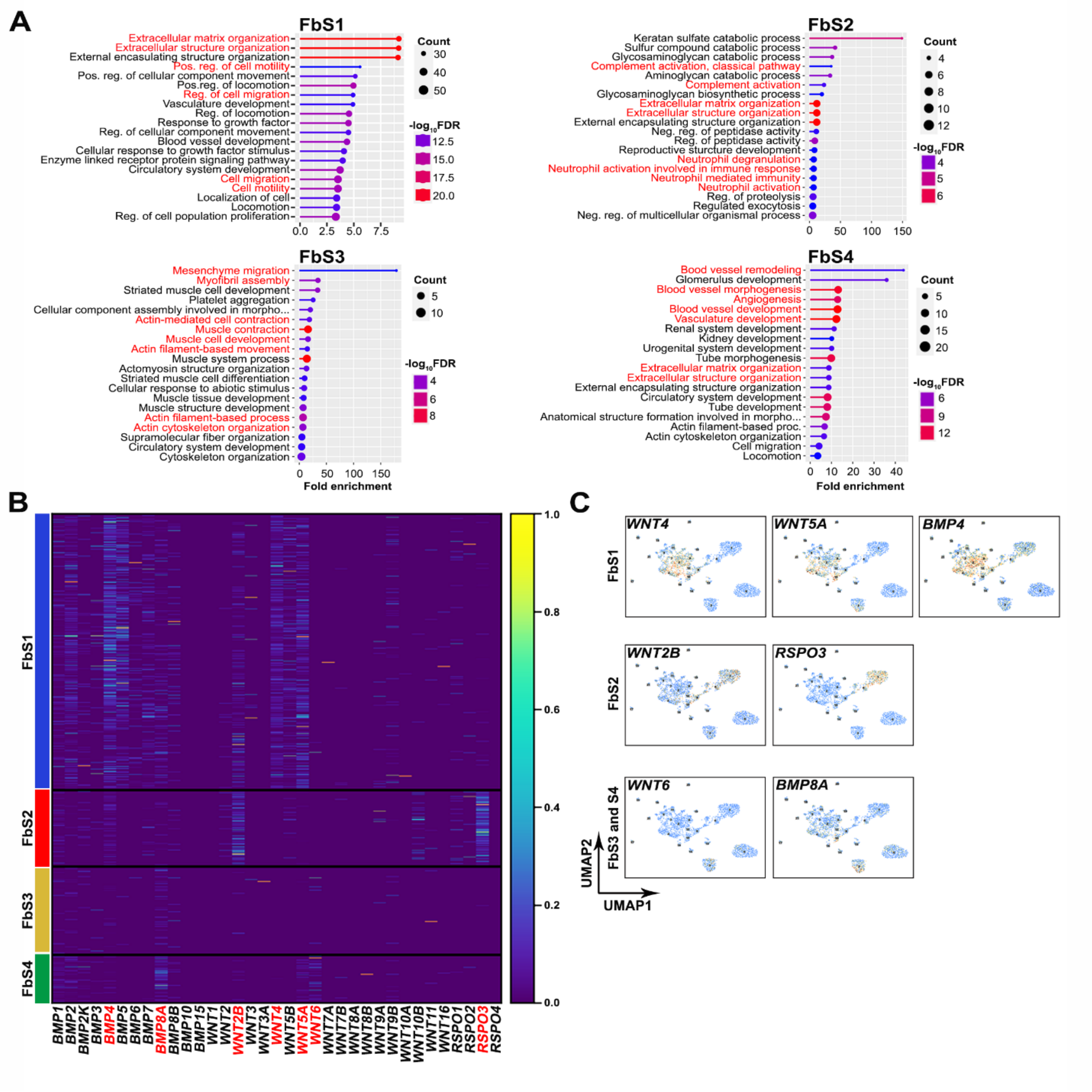

Supplementary Figure 5. Each fibroblast subset exhibits distinct genetic features and physiological functions. A, Bubble plots showing enriched pathways of DEGs in FbS1-S4 from scRNA-seq of fibroblasts, marked with fold enrichment, gene count and false discovery rate (FDR). Red-annotated pathways show exclusive characteristics of each fibroblast subset. **B**, Heatmap of differentially expressed WNTs, BMPs and RSPOs genes. **C**, UMAPs representing expression of selected WNTs, BMPs and RSPOs genes

marked with red in **B**, indicated by genes specific to each fibroblast subset.

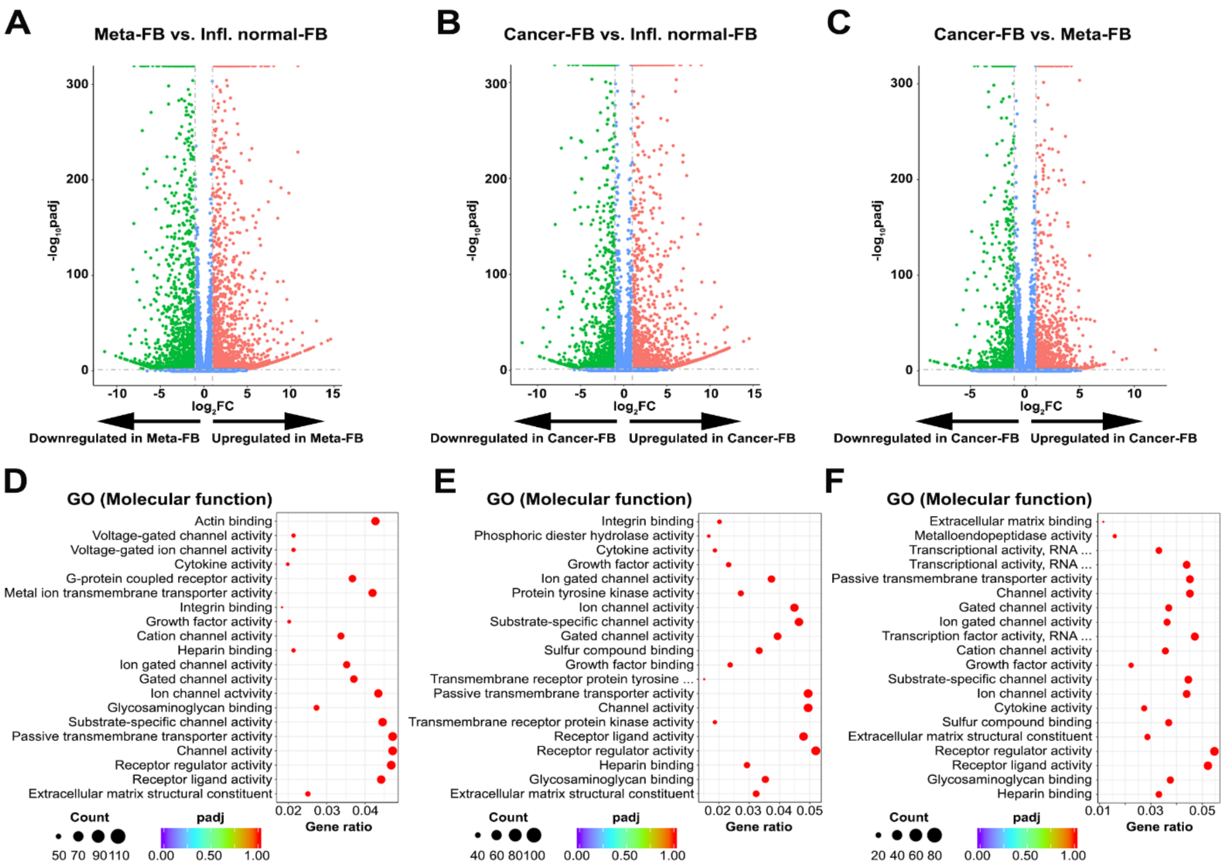

Supplementary Figure 6. **Bulk RNA-seq of patient-derived fibroblasts isolated from inflamed normal (Infl. normal-FBs), metaplastic (Meta-FBs) and cancer-bearing mucosae (Cancer-FBs) from the same patient shows distinct transcriptomic patterns. A-C**, Volcano plots showing DEGs from bulk RNA-seq data of Meta-FB versus Infl. normal-FB (A), Cancer-FB versus Infl. normal-FB (B) and Cancer-FB versus Meta-FB (C) samples. Adjusted p value (padj) and fold change (FC) are indicated. **D-F**, Bubble plots representing enriched pathways of DEGs from bulk RNA-seq data of Meta-FB versus Infl. normal-FB (D), Cancer-FB versus Infl. normal-FB (E) and Cancer-FB versus Meta-FB (F) samples, marked with gene count with ratio and adjusted p-value (padj).

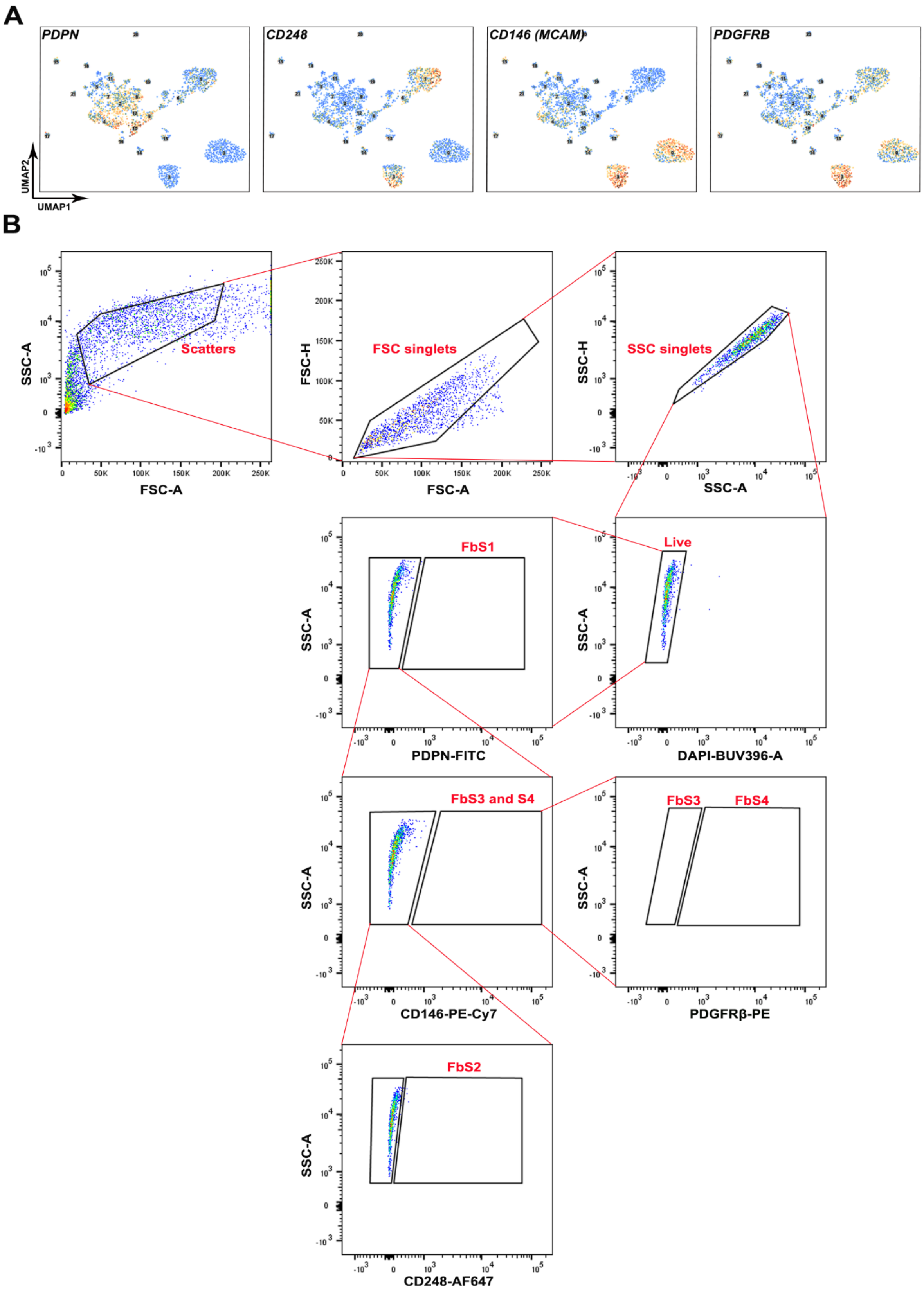
A
PDPN
CD248
CD146 (MCAM)
PDGFRB
UMAP2
UMAP1
B
SSC-A
FSC-A
Scatters
FSC-H
FSC singlets
SSC-H
SSC singlets
Live
DAPI-BUV396-A
FbS1
PDPN-FITC
FbS3 and S4
CD146-PE-Cy7
FbS3
FbS4
PDGFRβ-PE
FbS2
CD248-AF647

Supplementary Figure 7. **Gating strategy for fibroblast subset sorting from different mucosal pathology-derived fibroblasts. A**, UMAPs showing expression of genes encoding the surface markers used for FACS, indicated by genes specific to each cluster. **B**, Scheme of gating for fibroblast subsets sorting. DAPI-negative, live single cells are first sorted with FITC-conjugated PDPN. $PDPN^{hi}$ cells are considered as a FbS1, and then $PDPN^{lo}$ cells are gated on PE-Cy7-conjugated CD146 expression. Among $PDPN^{lo}$ $CD146^{lo}$ cells, CD248-expressing cells are regarded as a FbS2. $PDPN^{lo}$ $CD146^{hi}$ cells can be divided into PDGFRB low or high population, indicating FbS3 or S4, respectively.

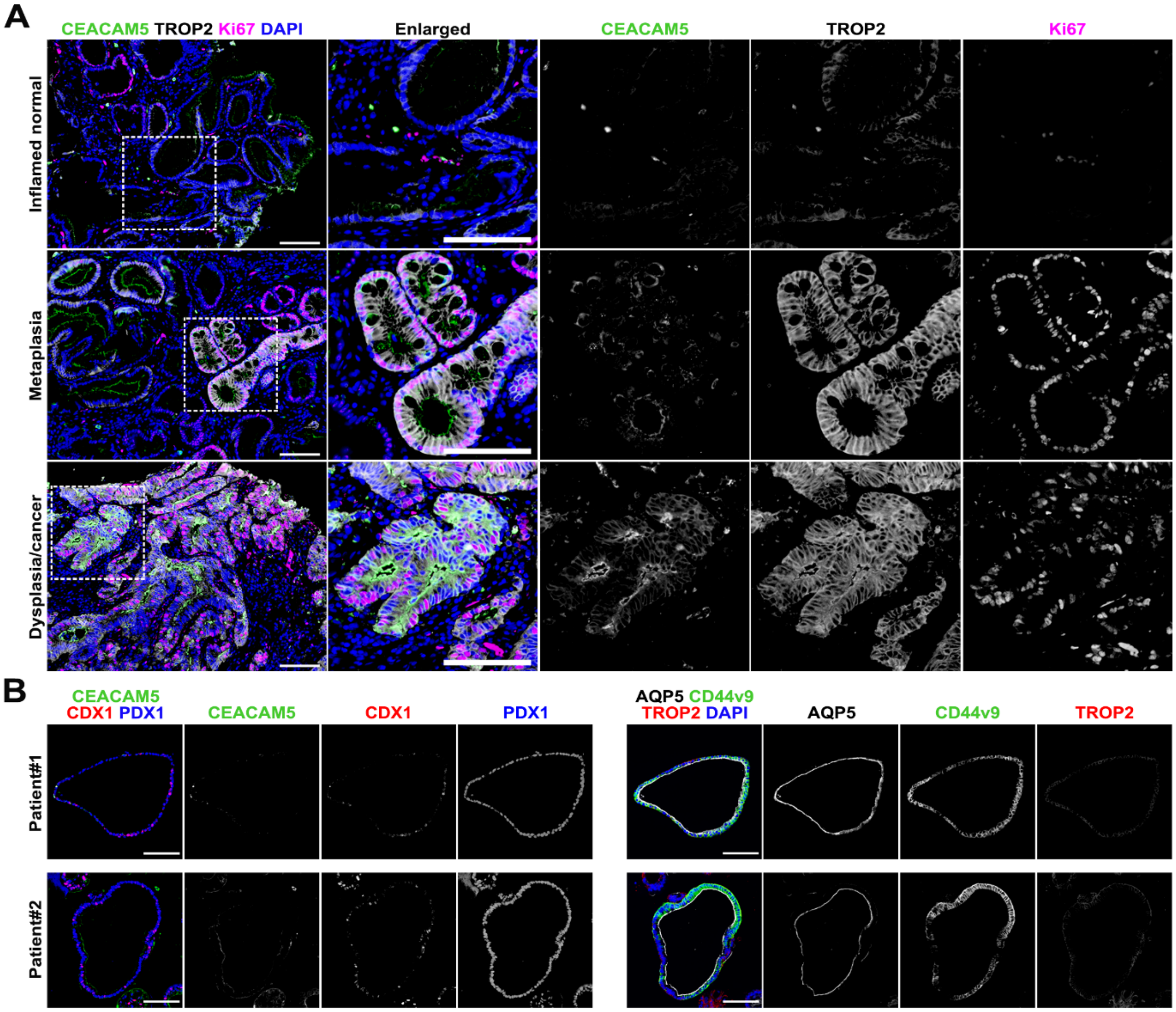

Supplementary Figure 8. **Characterization of patient tissues-derived gastroids. A**, Representative images of immunofluorescence staining for dysplasia marker, CEACAM5 and TROP2, and Ki67 in sections of inflamed normal, metaplasia or dysplasia/cancer human gastric tissues. Scale bar=100μm. **B**, Representative images of immunofluorescence staining for SPEM lineage marker AQP5, metaplasia marker CD44v9, dysplasia marker TROP2 (left panel), dysplasia marker CEACAM5, intestinal lineage marker CDX1 and antral/intestinal lineage marker PDX1 (right panel) in two different patients-derived gastroids (GOs). Scale bar=100μm.

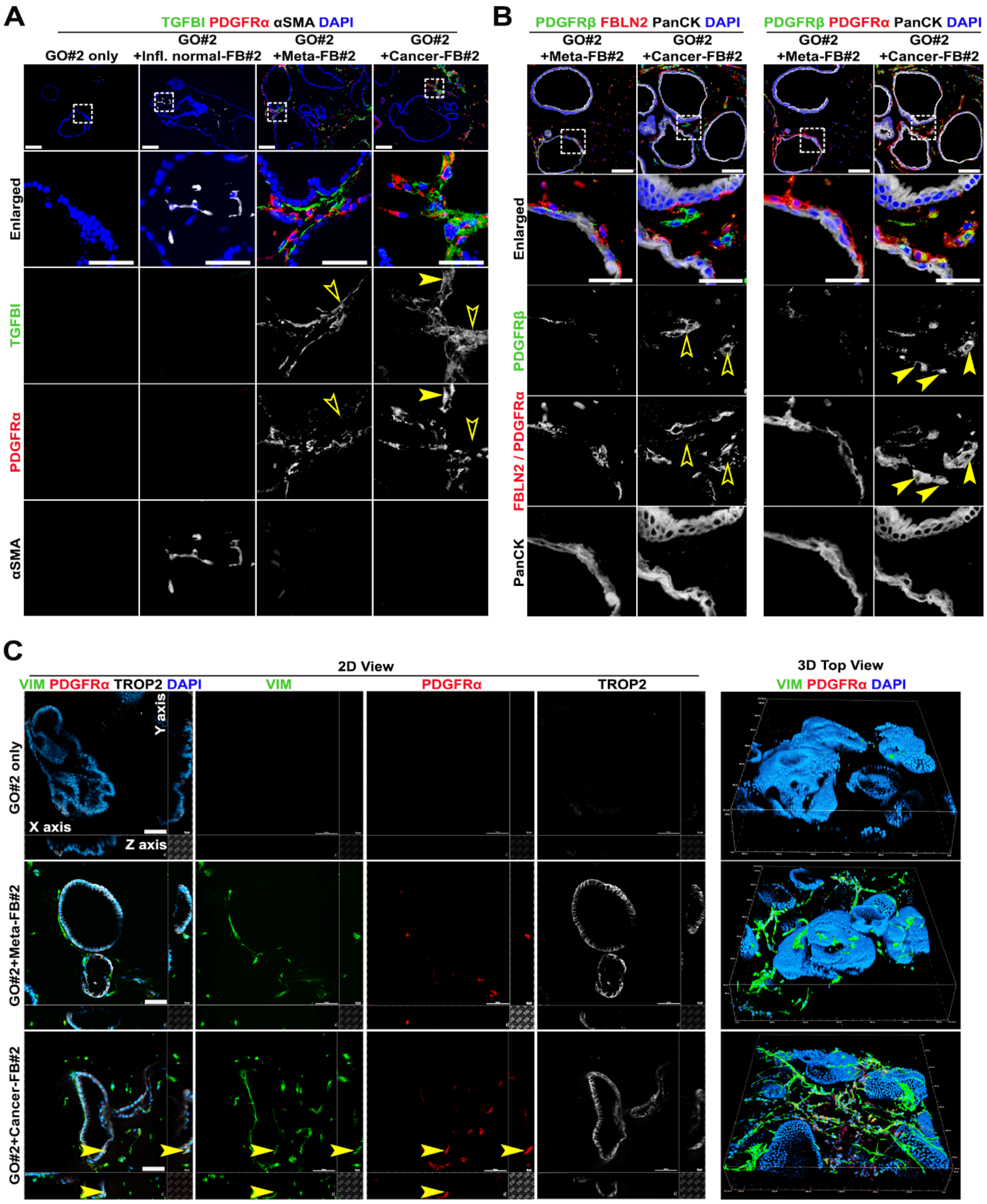

Supplementary Figure 9. Fibroblasts in 3-dimensional co-culture display

phenotypic characteristics of fibroblast subsets in gastric cancer patient tissue. **A**, Representative images of immunofluorescence for transforming growth factor beta induced (TGFBI) for fibroblasts, PDGFRα for FbS1 and αSMA for FbS3 with nuclear DAPI in inflamed normal-, metaplasia- or cancer-derived fibroblasts (Infl. normal-, Meta- or Cancer-FBs) cultured with metaplastic GOs. Note that αSMA^{+} FbS3 are more frequently observed in Infl. normal-FBs compared with others, and TGFBI expression can be observed in intracellular (arrowheads) and extracellular space (empty arrowheads) of Meta- and Cancer-FBs close to GOs. Scale bar=100 and 50μm for enlarged. **B**, Representative images of immunofluorescence for PDGFRα for FbS1, FBLN2 for FbS2, PDGFRβ for FbS4 and epithelial marker PanCK with nuclear DAPI in Meta- or Cancer-FBs cultured with metaplastic GOs. Note that PDGFRβ^{+} fibroblasts (green) are only notable in Cancer-FBs, and PDGFRβ expression is observed not in FBLN2^{+} (empty arrowheads) but in PDGFRα^{+} fibroblasts (arrowheads). Scale bar=100 and 50μm for enlarged. **C**, Representative images of whole mount staining of 3-dimensional co-culture. Two-dimensional optical sections (left panel) from a Z-stack acquisition (right panel) of whole-mounted metaplastic GOs co-cultured with fibroblasts representing direct contact of PDGFRα^{+} VIM^{+} fibroblasts (arrowheads) with GOs among VIM^{+} all fibroblasts interspersed between GOs. Scale bar=100μm (XY plan) and 20μm (XZ and YZ planes).

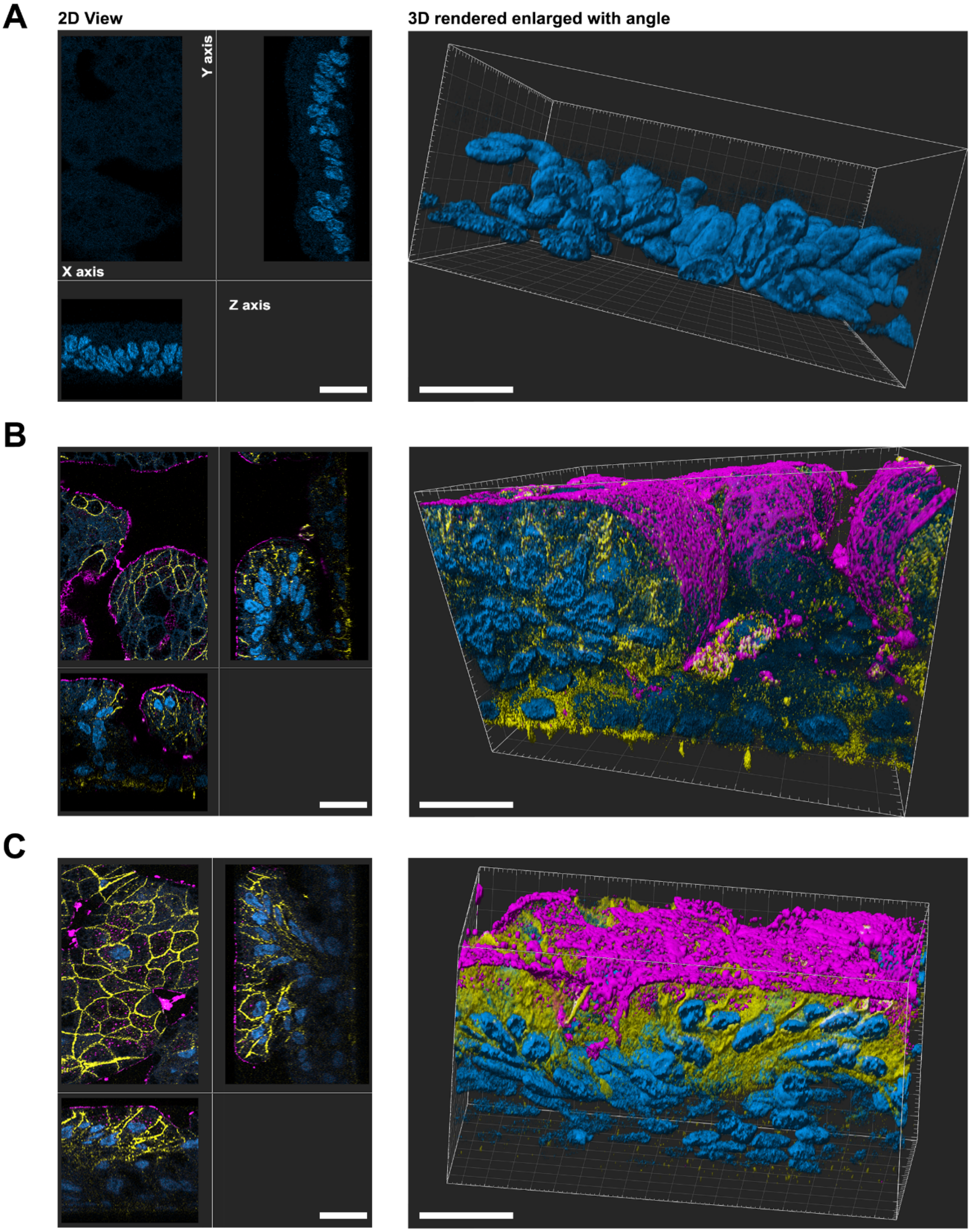

Supplementary Figure 10. Co-culture with Metaplasia-derived or Cancer-derived

fibroblasts induces expression of apical CEACAM5 and basolateral TROP2 in metaplastic gastroid cells in ALI condition. Whole-mount staining was performed on Transwell filters containing **(A)** metaplastic gastroid cells only, **(B)** metaplastic gastroid cells co-cultured with Meta-FBs or **(C)** metaplastic gastroid cells co-cultured with Cancer-FBs. Polypoid structures were observed in **B** and **C** co-expressing TROP2 (yellow) located at the basolateral membrane, and CEACAM5 (magenta) mainly located as puncta in the subapical cytoplasmic region. The metaplastic gastroid cells grown alone did not express CEACAM5 or TROP2 and did not show polypoid structures. Images were obtained with confocal microscopy. Left panels show top and side views of the samples and right panels show 3D rendered figures with nuclear DAPI staining (blue), all obtained using IMARIS software. Scale bar=20μm.

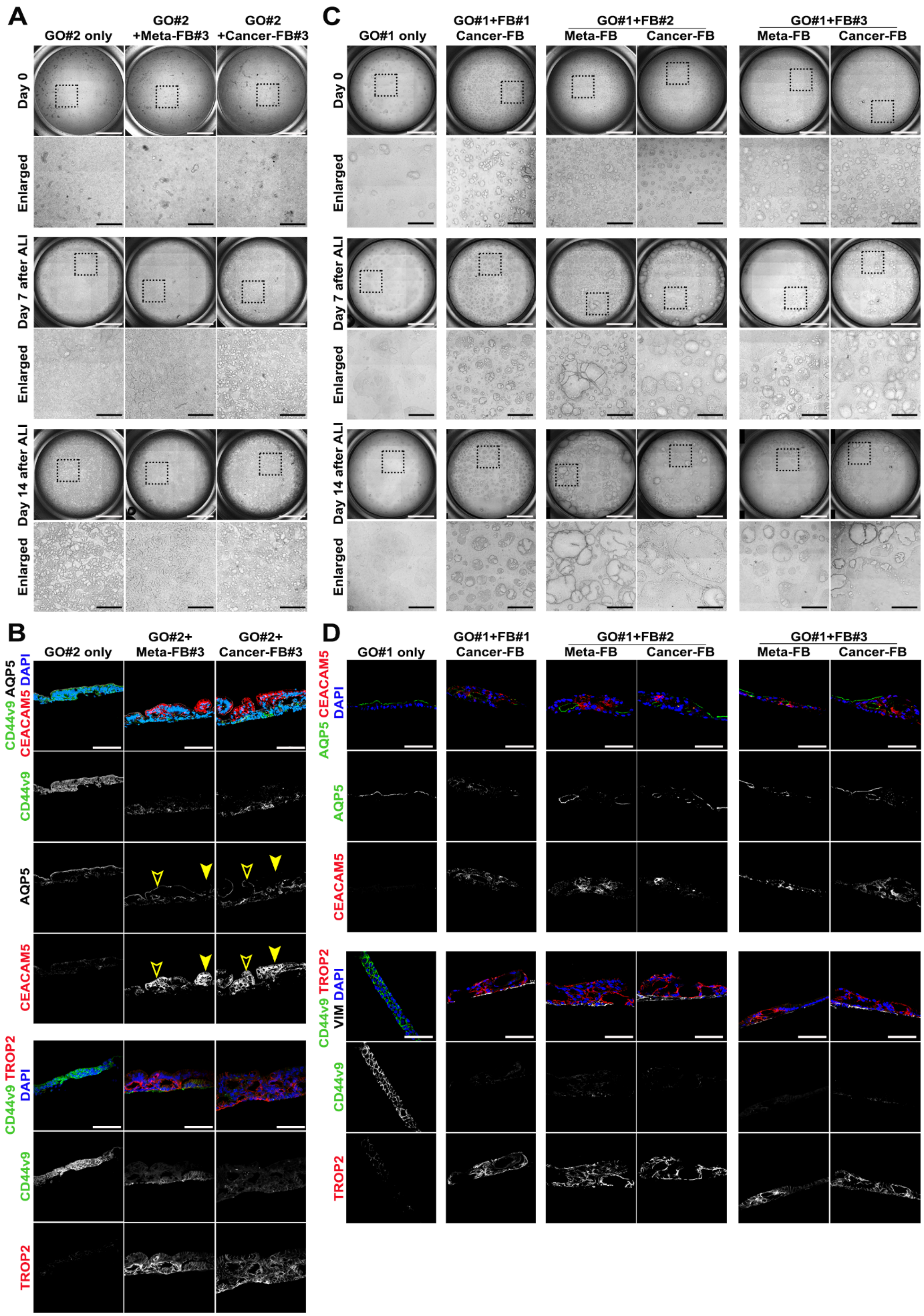
A
GO#2 only
GO#2 +Meta-FB#3
GO#2 +Cancer-FB#3
Day 0
Enlarged
Day 7 after ALI
Enlarged
Day 14 after ALI
Enlarged
C
GO#1 only
GO#1+FB#1 Cancer-FB
GO#1+FB#2
Meta-FB
Cancer-FB
GO#1+FB#3
Meta-FB
Cancer-FB
B
GO#2 only
GO#2+ Meta-FB#3
GO#2+ Cancer-FB#3
CD44v9 CEACAM5 AQP5 DAPI
CD44v9
AQP5
CEACAM5
CD44v9 TROP2 DAPI
CD44v9
TROP2
D
GO#1 only
GO#1+FB#1 Cancer-FB
GO#1+FB#2
Meta-FB
Cancer-FB
GO#1+FB#3
Meta-FB
Cancer-FB
AQP5 CEACAM5 DAPI
AQP5
CEACAM5
CD44v9 VIM TROP2 DAPI
CD44v9
TROP2

Supplementary Figure 11. **Metaplasia- or Cancer-derived fibroblasts from various patients can induce dysplastic progression in metaplastic gastroid cells from different patients. A**, Representative brightfield images at each time point of ALI co-culture of metaplastic gastroid (GO) cells from patient #2 with metaplasia- or cancer-derived fibroblasts (Meta- or Cancer-FBs) from patient #3 for 2 weeks. Scale bar=2000 and 500μm for enlarged. **B**, Representative images of immunofluorescence staining for metaplasia marker CD44v9, SPEM marker AQP5, dysplasia marker CEACAM5 (upper panel) and TROP2 (lower panel) with nuclear DAPI at 2 weeks after ALI co-culture. Note that CD44v9 expression is limited within the basal layer in co-cultured GO cells. $CEACAM5^{+}$ $AQP5^{-}$ (arrowheads) or $CEACAM5^{+}$ $AQP5^{+}$ (empty arrowheads) polyps in co-cultured GO cells are indicated. Scale bar=50μm. **C**, Representative brightfield images at each time point of ALI co-culture of metaplastic GO cells from patient#1 with Meta- or Cancer-FBs from patient #1, #2 or #3 for 2 weeks. Note that prominent multiple polyps in co-culture coalesced with each other over time. Scale bar=2000 and 500μm for enlarged. **D**, Representative images of immunofluorescence staining for SPEM marker AQP5 and dysplasia marker CEACAM5 (upper panel) or metaplasia marker CD44v9 and dysplasia marker TROP2 with fibroblast marker VIM (lower panel) and nuclear DAPI at 2 weeks after ALI co-culture. Compared with the gastroid cells only condition, polypoid protrusion from the base was increased in gastroid cells co-cultured with either metaplasia- or cancer-derived fibroblasts during the entire period of ALI culture, accompanied by increased expression of dysplastic markers, CEACAM5 and TROP2, and decreases in metaplastic markers, AQP5 and CD44v9. Scale bar=2000and 500μm for enlarged.

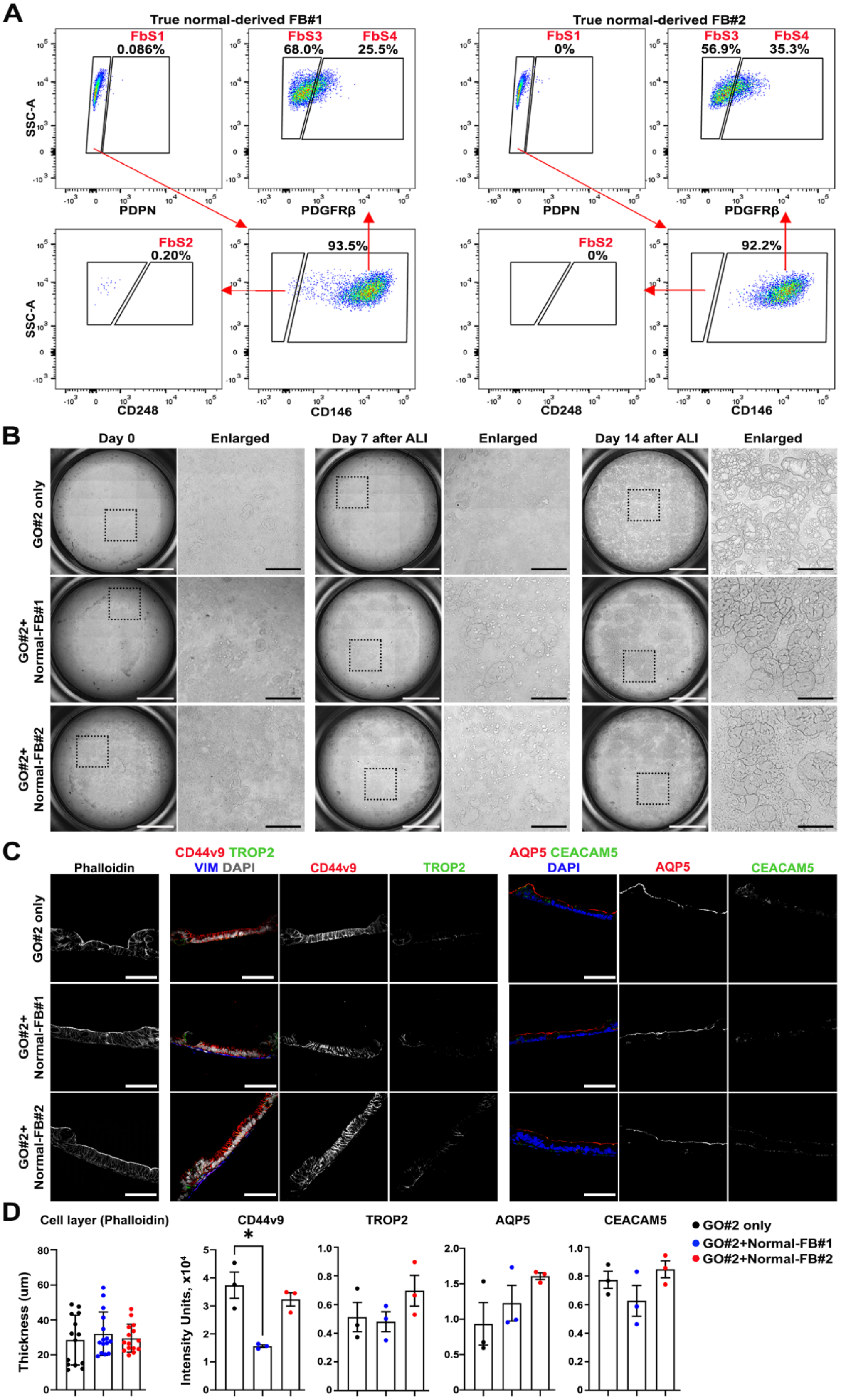

A
True normal-derived FB#1
True normal-derived FB#2
FbS1
0.086%
FbS3
68.0%
FbS4
25.5%
FbS1
0%
FbS3
56.9%
FbS4
35.3%
SSC-A
PDPN
PDGFRβ
FbS2
0.20%
93.5%
FbS2
0%
92.2%
CD248
CD146
B
Day 0
Enlarged
Day 7 after ALI
Enlarged
Day 14 after ALI
Enlarged
GO#2 only
GO#2+ Normal-FB#1
GO#2+ Normal-FB#2
C
Phalloidin
CD44v9 TROP2
VIM DAPI
CD44v9
TROP2
AQP5 CEACAM5
DAPI
AQP5
CEACAM5
GO#2 only
GO#2+ Normal-FB#1
GO#2+ Normal-FB#2
D
Cell layer (Phalloidin)
CD44v9
TROP2
AQP5
CEACAM5
GO#2 only
GO#2+Normal-FB#1
GO#2+Normal-FB#2
Thickness (um)
Intensity Units, x10⁴
*

Supplementary Figure 12. **True normal gastric mucosa-derived fibroblasts, mainly composed of FbS3 and FbS4, do not induce dysplastic progression of metaplastic gastroid cells. A**, FACS plots showing the proportion of four fibroblast subsets in two different true normal-derived fibroblast lines, according to the gating strategy described in **Supplementary Fig. 6B**. **B**, Representative brightfield images of co-cultured metaplastic gastroid (GO) cells with true normal-derived fibroblasts at each time point for 2 weeks. Scale bar=2000 and 500μm for enlarged. **C**, Representative images of immunofluorescence staining for Phalloidin (left panel), metaplasia marker CD44v9, dysplasia marker TROP2, fibroblast marker VIM (middle panel), SPEM marker AQP5 and dysplasia marker CEACAM5 (right panel) with nuclear DAPI. Co-culture with true normal-derived fibroblasts did not induce any changes in the expression of metaplasia or dysplasia markers in metaplastic GOs. Scale bar=100μm. **D**, Quantifications of thickness of GO cell layer determined by Phalloidin staining and extent of protein expression measured by intensity units. Data are presented as mean ± SD (n= 3 of different sections). *, $P < 0.05$.

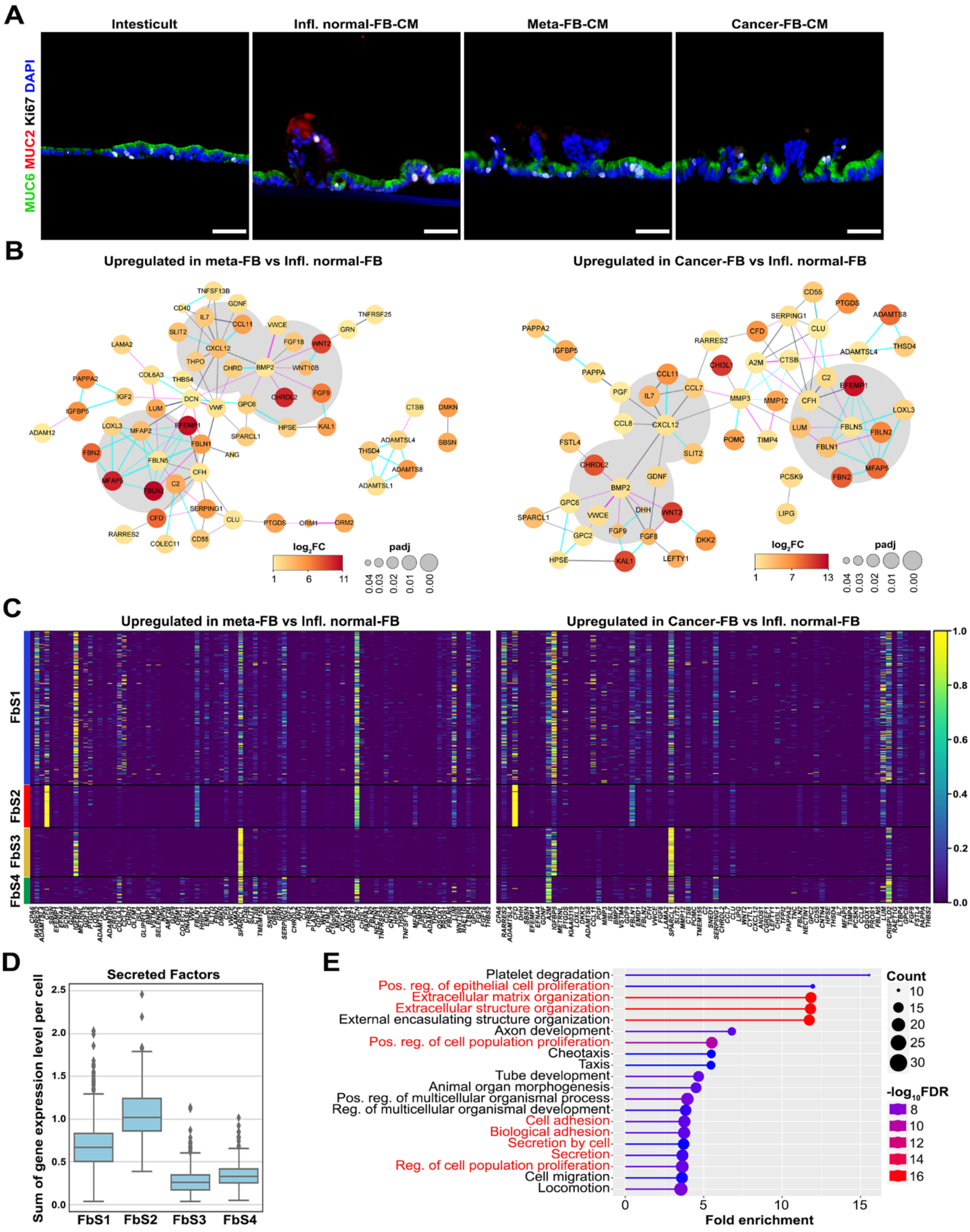

A
Intesticult
Infl. normal-FB-CM
Meta-FB-CM
Cancer-FB-CM
MUC6 MUC2 Ki67 DAPI
B
Upregulated in meta-FB vs Infl. normal-FB
Upregulated in Cancer-FB vs Infl. normal-FB
log2FC
padj
C
Upregulated in meta-FB vs Infl. normal-FB
Upregulated in Cancer-FB vs Infl. normal-FB
FbS1
FbS2
FbS3
FbS4
D
Secreted Factors
Sum of gene expression level per cell
FbS1
FbS2
FbS3
FbS4
E
Platelet degradation
Pos. reg. of epithelial cell proliferation
Extracellular matrix organization
Extracellular structure organization
External encasulating structure organization
Axon development
Pos. reg. of cell population proliferation
Cheotaxis
Taxis
Tube development
Animal organ morphogenesis
Pos. reg. of multicellular organismal process
Reg. of multicellular organismal development
Cell adhesion
Biological adhesion
Secretion by cell
Secretion
Reg. of cell population proliferation
Cell migration
Locomotion
Fold enrichment
Count
-log10FDR

Supplementary Figure 13. **Fibroblast subsets differentially express secreted factor-encoding genes that can alter epithelial behaviors. A**, Representative images of immunofluorescence staining for gastric type mucin 6 (MUC6), intestinal type mucin 2 (MUC2) and proliferation marker Ki67 with nuclear DAPI. Scale bar=50μm. **B**, Protein-protein interaction networks of 79 or 96 upregulated genes in bulk RNA-seq data of metaplasia- (Meta-) or cancer-derived fibroblasts compared with inflamed normal (infl. normal)-derived fibroblasts. Edges were constructed based on the curated databases (cyan), previous experiments (magenta) or textmining (grey). Three grey-colored circles indicate hubs commonly observed in both networks. Networks were constructed using Cytoscape software 3.9.0. **C**, Heatmaps of the secreted factor-encoding genes investigated in **B** showing the distinctive expression pattern of fibroblast subsets from scRNA-seq data. Rows indicate single cells. **D**, A box and whiskers plot showing sum of the normalized expression level of genes listed in **C** per each fibroblast subset. **E**, Bubble plots representing enriched terms (Gene Ontology; biological process) in genes listed in **C**, marked with gene count, fold enrichment and false discovery rate (FDR).

Supplementary Table 1. Patient characteristics, pathological findings and use for scRNA sequencing, gastroid preparation, fibroblast preparation and immunostaining.

Pt	Sex	Age	Dx	Differentiation	Stage	Tumor Size (maximal dimension)	Tumor Location	Active H. pylori Infection	Additional pathological findings	scRNA-seq	Gastroid	fibroblasts	Immune-staining
1	M	85	Gastric adenocarcinoma, intestinal type	G2: moderately	pT3N2	11.5 cm	Body	present	Extensive atrophic gastritis and intestinal metaplasia		X	X	X
2	M	72	Gastric adenocarcinoma, intestinal type	G2: moderately	pT2N1	4.5 cm	Antrum	present	Chronic active gastritis, intestinal metaplasia with active H. pylori infection		X	X	X
3	M	86	Gastric adenocarcinoma, intestinal type	G3: Poorly	pT2N0	2.5 cm	Antrum	absent	Extensive intestinal metaplasia with dysplasia	X	X	X	X
4	F	84	Diffuse-type adenocarcinoma; poorly cohesive carcinoma (signet-ring cell carcinoma)	G3: Poorly	pT4N0	2.9 cm	Body	absent	Atrophic gastritis with extensive intestinal metaplasia	X			
5	F	83	Gastric adenocarcinoma, intestinal type	G1: Well	pT1aN0	6.0 cm	Antrum	absent	Extensive intestinal metaplasia with low and high grade dysplasia	X			X
6	M	62	Diffuse-type adenocarcinoma; poorly cohesive carcinoma (signet-ring cell carcinoma)	G3: Poorly	pT2N0	3.2 cm	Antrum	present	Extensive intestinal metaplasia with H. Pylori type gastritis	X			X
7	M	77	Diffuse-type adenocarcinoma; poorly cohesive carcinoma (signet-ring cell carcinoma)	G3: Poorly	pT3N3a	2.7 cm	Body	present	Intestinal metaplasia, H. pylori-type gastritis, low-grade dysplasia; associated with the invasive carcinoma, focal areas suggestive of foveolar-type dysplasia	X	X	X	X

Supplementary Table 3. List of primary antibodies for immunofluorescence staining.

Antibody	Species	Vender, Catalog#	Dilution
AQP5	Rabbit	Sigma, HPA065008	1:500
CD44v9	Rat	Cosmo Bio, LKG-M001	1:15,000
CDX1	Rabbit	Thermo Fisher, PA5-23056	1:300
CEACAM5	Mouse	Abclonal, A18131	1:1,000
CLDN7	Rabbit	Thermo Fisher, 34-9100	1:1,000
FBLN2	Rabbit	Sigma, HPA001934	1:500
Ki-67	Rat	LSBio, LS-C338537	1:500
MUC2	Rabbit	SantaCruz, sc-15334	1:200
MUC6	Mouse	Abcam, ab216017	1:200
P120	Mouse	BD Biosciences, 610133	1:100
PDGFRα	Rabbit	Cell Signaling, 3164S	1:200
PDGFRβ	Rabbit	Thermo Fisher, MA5-15143	1:200
PDX1	Guinea Pig	Gift from Chris Wright, VUMC	1:500
TGFBI	Rabbit	Sigma, HPA008612	1:500
TROP2	Goat	R&D Systems, AF650	1:500
Vimentin	Mouse	Sigma, V6630	1:500
AQP5, Alexa Fluor 488 conjugated	Mouse	SantaCruz, sc-514022	1:100
PanCK, Alexa Fluor 790 conjugated	Mouse	Novus, NBP2-33200AF750	1:100
PCNA, Alexa Fluor 488 conjugated	Mouse	SantaCruz, sc-56	1:50
Phalloidin, iFluor 488 conjugated		Abcam, ab176753	1:100
αSMA, Alexa Fluor 546 conjugated	Rabbit	Cell Signaling, 60839S	1:500

Supplementary Table 4. List of primary antibodies for fluorescence-activated cell sorting (FACS).

Antibody	Species	Source	Dilution
PDPN, FITC conjugated	Mouse	Novus Biologicals, NBP2-54347F	1:200
CD248, Alexa Fluor 647 conjugated	Rabbit	Bioss Antibodies, bs-2101R-A647	1:200
CD146, PE-Cy7 conjugated	Mouse	Thermo Fisher, 25-1469-42	1:500
PDGFRβ, PE conjugated	Mouse	Thermo Fisher, MA1-10102	1:50
